# Comprehensive analysis of the effects of *P4ha1* and *P4ha2* deletion on post-translational modifications of fibrillar collagens in mouse skin

**DOI:** 10.3389/fcell.2025.1527839

**Published:** 2025-02-28

**Authors:** Vivek Sarohi

**Affiliations:** School of Biosciences and Bioengineering (SBB), Indian Institute of Technology (IIT)- Mandi, Mandi, India

**Keywords:** collagen PTMs, prolyl 4-hydroxylation, prolyl 3-hydroxylation, lysyl 5-hydroxylation, O-glycosylation

## Abstract

**Introduction:**

Collagens, the most abundant proteins in mammals, play pivotal roles in maintaining tissue structure, functions, cell-to-cell communication, cellular migration, cellular behavior, and growth. Structures of collagens are highly complex due to the presence of dynamic post-translational modifications (PTMs), such as hydroxylations (on prolines and lysine residues) and O-glycosylation (on hydroxylysines) enzymatically catalyzed during biosynthesis in the endoplasmic reticulum. Collagen PTMs are essential for maintaining structural stability, elasticity, and different functions of collagens. The most prevalent modification in fibrillar collagens is prolyl 4-hydroxylation catalyzed by collagen prolyl 4-hydroxylases (C-P4Hs). Prolyl 4-hydroxylation on collagens plays a critical role in collagen biosynthesis, thermostability, and cell-collagen interactions. Collagens are large proteins. Different regions of collagen perform different functions, so the presence or absence of a PTM on a particular collagen site can affect its functioning. However, comprehensive site-specific identification of these PTMs on fibrillar collagen chains of mice skin has not been performed yet. Furthermore, the effects of prolyl 4-hydroxylase alpha 1 (P4HA1) and P4HA2 on 3-hydroxyproline, 5-hydroxylysine, and O-glycosylation sites of fibrillar collagen chains have not yet been explored.

**Methodology:**

This study presents a comprehensive PTM analysis of fibrillar collagen chains extracted from the skin of different mutants of C-P4Hs (*P4ha1* ^*+/−*^; *P4ha2−/−*, *P4ha1* ^*+/+*^; *P4ha2−/−*, *P4ha1* ^*+/−*^; *P4ha2* ^*+/−*^, *P4ha1* ^*+/+*^; *P4ha2* ^*+/−*^) and wild-type mice. In this study, proteomics-based comprehensive PTM site identification by MS2 level ions from raw mass spectrometry data was performed, and MS1-level quantification was performed for PTM occupancy percentage analysis.

**Results and discussion:**

A total of 421 site-specific PTMs were identified on fibrillar collagen chains (COL1A1, COL1A2, and COL3A1) extracted from wild-type mice skin. A total of 23 P4HA1-specific and seven P4HA2-specific 4-hydroxyproline sites on fibrillar collagen chains were identified. Moreover, it was found that the *P4ha1* and *P4ha2* deletion can affect the 3-hydroxyproline occupancy percentages in mice skin. Interestingly, increased levels of lysyl 5-hydroxylation were detected upon partial deletion of *P4ha1* and full deletion of *P4ha2*. These findings show that the effects of deletion of prolyl 4-hydroxylases are not limited to less 4-hydroxylation on some specific proline sites, but it can also modulate the prolyl 3-hydroxylation, lysyl 5-hydroxylation, and O-glycosylation occupancy percentages in the fibrillar collagen chains in a site-specific manner.

## 1 Introduction

Collagens are the most abundant component of the extracellular matrix (ECM). Collagens provide an attachment surface to the cells in the body and maintain the structural stability and elasticity of all tissues (Gelse et al., 2003). Collagens play important roles in cellular functions, including cell-to-cell communication, cell migration, and cell growth by interaction with cell-surface receptors (integrins, discoidin domain receptor, glycoprotein VI, and FC gamma receptor) (Leitinger and Hohenester, 2007; Borza et al., 2018; Sarohi et al., 2022a; Farndale et al., 2023). Collagens are very dynamic in structure. Collagens are heavily decorated with PTMs during biosynthesis in the endoplasmic reticulum (ER) prior to helix formation. PTMs are required for proper folding, stability, and functioning of the collagens (Takaluoma et al., 2007; Vranka et al., 2010; Pokidysheva et al., 2014; Terajima et al., 2014; Montgomery et al., 2018; Morello, 2018; Rappu et al., 2019; Tonelli et al., 2020).

Collagen PTMs are site-specifically catalyzed by collagen-modifying enzymes. Collagen prolyl 4-hydroxylases (C-P4Hs), that is, prolyl 4-hydroxylase alpha 1 (P4HA1), P4HA2, and P4HA3, catalyze 4-hydroxylation on prolines present in collagen chains. The 3-hydroxylation on proline is further catalyzed by prolyl 3-hydroxylase 1 (P3H1), P3H2, and P3H3 in the collagen chains. The lysine sites present in the collagens can also be 5-hydroxylated. Lysyl hydroxylation in the collagen is catalyzed by lysyl hydroxylase 1 (LH1), LH2, and LH3 encoded by procollagen-lysine,2-oxoglutarate 5-dioxygenases (*Plods*). Furthermore, 5-hydroxylysines in collagen chains can be O-glycosylated to galactosyl-hydroxylysine (G-HyK) and glucosylgalactosyl-hydroxylysine (GG-HyK). G-HyK on 5-hydroxylysines is catalyzed by procollagen galactosyltransferases (COLGALTs), and GG-HyK is primarily catalyzed by LH3. One of the most well-studied PTM in the fibrillar collagen chain is prolyl 4-hydroxylation (Weiss and Klein, 1969; Risteli and Kivirikko, 1974; Chu et al., 1997; Nishi et al., 2005; Sipila et al., 2018; Rappu et al., 2019). The role of prolyl 4-hydroxylases has been studied in thermal stability and in adverse ECM remodeling leading to pathophysiological complications, such as fibrosis and cancer progression (Vasta and Raines, 2018; Xiong et al., 2018; Li et al., 2021; Shi et al., 2021).

The two most abundant C-P4H forms in mice skin are P4HA1 and P4HA2 (Salo et al., 2024). The P4HA1 enzyme is essential for the functioning of the mouse body, and complete deletion of *P4ha1* (*P4ha1−/−*) is embryonically lethal (Sipila et al., 2018; Tolonen et al., 2022). However, mice with a partial deletion of *P4ha1* (*P4ha1* ^*+/−*^) are viable, and mice with partial (*P4ha2* ^*+/−*^) or complete (*P4ha2−/−*) deletion of *P4ha2* are also viable with no apparent phenotype (Sipila et al., 2018; Tolonen et al., 2022). Interestingly, mice with a partial deletion of *P4ha1* (*P4ha1* ^*+/−*^) and partial deletion of *P4ha2* (*P4ha2* ^*+/−*^) also survive. However, a comprehensive analysis of the effects of *P4ha1* and *P4ha2* deletion on collagen PTMs, including 3-hydroxyproline, 5-hydroxylysine, and O-glycosylation, has not yet been explored. Moreover, comprehensive site-specific identification of collagen PTMs in fibrillar collagen chains has not been performed. In this study, PTM analysis was performed to study the effects of deletion of *P4ha1* and *P4ha2* on site-specific modifications of fibrillar collagen chains (COL1A1, COL1A2, and COL3A1) extracted from the wild-type and *P4ha1* and *P4ha2* deletion mutant (*P4ha1* ^*+/−*^; *P4ha2−/−*, *P4ha1* ^*+/+*^; *P4ha2−/−*, *P4ha1* ^*+/−*^; *P4ha2* ^*+/−*^, *P4ha1* ^*+/+*^; *P4ha2* ^*+/−*^) mice. This study utilizes the publicly available raw mass spectrometry data #PXD008802 (Sipila et al., 2018) of mice skin fibrillar collagen extracted from wild-type mice and different deletion mutants of *P4ha1* and *P4ha2*.

## 2 Methods

### 2.1 Mass spectrometry data resource

The publicly available proteomic dataset #PXD008802 submitted by Sipila et al. (2018) in ProteomeXchange was utilized in this study. This dataset contains raw mass spectrometry data (54 files) of fibrillar collagen extracted from the skin of prolyl 4-hydroxylase mutant and wild-type mice. There are 12 raw mass spectrometry files present for the *P4ha1* ^*+/−*^; *P4ha2−/−* mice group, 14 raw files for *P4ha1* ^*+/+*^; *P4ha2−/−*, 10 raw files for *P4ha1* ^*+/−*^; *P4ha2* ^*+/−*^, eight raw files for *P4ha1* ^*+/+*^; *P4ha2* ^*+/−*^, and 10 raw files for the wild-type mice group. All the biological replicates had a technical duplicate. This proteomic dataset acquisition was performed using a nanoflow HPLC system (Easy-nLC1000, Thermo Fisher Scientific) coupled with a Q-Exactive mass spectrometer from ThermoFisher, as described earlier (Sipila et al., 2018).

### 2.2 Proteomic data analysis

In this study, 54 raw mass spectrometry files from dataset #PXD008802 were analyzed. The MyriMatch (Tabb et al., 2007) search engine was utilized for proteomic database searches. A two-step MyriMatch database search strategy (general search followed by PTM-specific search) was employed for in-depth analysis of site-specific collagen PTM (hydroxylation of prolines and lysines and O-glycosylation of hydroxylysines) present on fibrillar collagen chains of mice skin.

#### 2.2.1 General MyriMatch database search

First, a general MyriMatch (Tabb et al., 2007) database search was performed on the *Mus musculus* database downloaded from Uniprot.org (downloaded on 4-Dec-2021) containing 17,090 entries. In general search, the tolerance for precursors was set at ±10 ppm, and for fragment ions, it was allowed up to ±20 ppm. Fully tryptic peptides were considered for database search with missed cleavages allowed up to a maximum of two per peptide. Carbamidomethylation (+57.0236) on cysteine was used as static modification, and methionine oxidation (+15.994916) along with hydroxyproline (+15.994916) were used as dynamic modifications (Sarohi et al., 2022b). The maximum dynamic modifications per peptide were set to a maximum of four per peptide. After the completion of the MyriMatch general database search, the results were imported to IDPICKER (Ma et al., 2009) for parsimonious protein grouping and controlling FDR. The FDR was controlled at <1% at peptide-spectrum matches (PSMs), peptide, and protein group levels. Then, this FDR-controlled list of identified proteins was exported from IDPicker as subset fasta. This subset fasta was utilized for an in-depth collagen PTM-specific database search.

#### 2.2.2 MyriMatch PTM-specific database search

In the PTM-specific subset fasta database search using MyriMatch, precursor and fragment ion mass tolerance was kept at 10 ppm and 20 ppm, respectively (Sarohi et al., 2022b). Carbamidomethylation (+57.0236) of cysteine was used as static modification, and oxidation (+15.994916) of methionine was used as dynamic modification. Oxidation (+15.994916) of proline was used as a dynamic modification for the detection of hydroxyprolines in the full-length collagen chains. Further motif-based following dynamic modifications were also included: 3 prolyl-hydroxylation (GP!P! 15.994916), lysyl hydroxylation (GXK! 15.994916), galactosyl-hydroxylysine (GXK! 178.047738), and glucosylgalactosyl-hydroxylysine (GXK! 340.100562) (Sarohi et al., 2022b). A maximum of 10 modifications were allowed per peptide. A maximum number of four missed cleavages per fully tryptic peptide was allowed. After completion of the MyriMatch PTM-specific database search, the result files were further grouped for PSM matches, peptide, and protein group identification using IDPicker with <1% FDR (for PSM, peptide, and protein IDs). Identification of 3-hydroxyprolines was only considered if a proline residue was found to be hydroxylated at the “X” position of a “-Xaa-HyP-Gly” motif in the collagen chains. Furthermore, IDPicker and pLabel (Wang et al., 2007) were used for the identification, manual inspection, analysis, and validation of peptide-spectrum matches (PSMs) for assigning site-specific PTMs on fibrillar collagen chains.

#### 2.2.3 MS1 level quantitation of the occupancy percentage of site-specific collagen PTMs using Skyline

The results files of the MyriMatch PTM-specific database search were parsed through Peptide Prophet (TPP pipeline module) (Pedrioli, 2010) for probability scoring between 0 and 1. Parsed MyriMatch results were used to build the spectral library in the Skyline (MacLean et al., 2010). The spectral library was utilized for the extraction of MS1 intensities of different unmodified and modified forms of collagen peptides from the raw mass spectrometry. For the occupancy percentage calculation, MS1 intensities of valid modified (with a particular modification site) peptides and respective unmodified peptides were extracted using Skyline.

### 2.3 Statistical analysis

A two-tailed Student’s test was applied for the statistical analysis of the occupancy percentage of site-specific PTMs. A p-value of <0.05 was considered statistically significant in all tests.

## 3 Results

Skin is one of the most collagen-rich tissues of the mouse. The high abundance of collagen in the skin provides the opportunity for in-depth collagen PTM analysis. In this study, site-specific identification of PTMs was performed on fibrillar collagen chains extracted from wild-type mice skin. Additionally, site-specific PTM quantitation was performed to assess the effects of *P4ha1* and *P4ha2* deletion on fibrillar collagen PTM networks.

### 3.1 Identification of site-specific collagen PTMs in fibrillar collagens extracted from wild-type mouse skin

In this study, site-specific collagen PTMs, that is, 4-hydroxyproline (4-HyP), 3-hydroxyproline (3-HyP), hydroxyproline (HyP), 5-hydroxylysine (HyK), galactosyl-hydroxylysine (G-HyK), and glucosylgalactosyl-hydroxylysine (GG-HyK) sites were identified on COL1A1, COL1A2, and COL3A1 chains extracted from the wild-type mice skin (Table 1). Hydroxyproline present on the Yaa position of the -Xaa-Yaa-Gly- motif was considered as 4-hydroxyproline. Hydroxyproline present on the Xaa position of the -Xaa-4HyP-Gly- motif was considered as 3-hydroxyproline. Sites where hydroxyproline is present on the Xaa position of the -Xaa-Yaa-Gly- motif and Yaa is not a proline/hydroxyproline were labeled as only “hydroxyproline” sites in this study. However, there is a high probability that these sites are also 4-hydroxyproline sites (Van Huizen et al., 2019). Hydroxylysine present on the Yaa position of -Xaa-Yaa-Gly was considered as 5-hydroxylysine (Perdivara et al., 2013). The O-glycosylation was identified based on the mass of monosaccharide (G-HyK) and disaccharide (GG-HyK) (Perdivara et al., 2013; Yamauchi et al., 2019; Sarohi et al., 2022b).

**TABLE 1:** Site-specific collagen PTMs identified on fibrillar collagen chains extracted from wild-type mouse skin. Collagen PTMs, that is, 4-hydroxyproline (4-HyP), 3-hydroxyproline (3-HyP), hydroxyproline (HyP), 5-hydroxylysine (HyK), galactosyl-hydroxylysine (G-HyK), and glucosylgalactosyl-hydroxylysine (GG-HyK), are listed under the “Modifications” column in the table. The -Xaa-Yaa-Gly- motif of collagen PTMs is represented under the “Motif” column, where a one-letter code for three amino acids (X position amino acid, Y position amino acid, and glycine) is written for every collagen PTM that was identified separately in COL1A1, COL1A2, and COL3A1 chains. Under the “Site” column, the specific site (number of that amino acid in the full-length protein) that is identified to have a specific PTM is written in Table 1.

COL1A1			COL1A2			COL3A1		
Modification	Motif	Site	Modification	Motif	Site	Modification	Motif	Site
4-HyP	VPG	167	HyP	GPG	96	4-HyP	LPG	244
HyP	PMG	169	4-HyP	PPG	108	4-HyP	PPG	247
4-HyP	LPG	179	4-HyP	APG	114	GG-HyK	IKG	250
4-HyP	PPG	182	4-HyP	EPG	126	4-HyP	MPG	256
4-HyP	PPG	194	4-HyP	EPG	129	HyK	EKG	274
4-HyP	EPG	197	4-HyP	SPG	144	4-HyP	APG	280
4-HyP	EPG	200	4-HyP	HPG	153	HyK	LKG	283
4-HyP	PPG	212	4-HyP	KPG	156	4-HyP	LPG	289
4-HyP	PPG	215	4-HyP	RPG	159	4-HyP	APG	298
4-HyP	RPG	230	4-HyP	FPG	174	4-HyP	RPG	310
4-HyP	PPG	236	4-HyP	TPG	177	4-HyP	LPG	313
4-HyP	LPG	245	4-HyP	LPG	180	4-HyP	QPG	331
4-HyP	LPG	251	HyK	FKG	183	4-HyP	PPG	334
GG-HyK	MKG	254	HyK	VKG	186	4-HyP	PPG	337
HyK	AKG	266	HyK	LKG	195	4-HyP	FPG	343
HyK	PKG	275	4-HyP	QPG	198	4-HyP	SPG	346
G-HyK	PKG	275	HyK	VKG	204	4-HyP	SPG	358
HyP	PAG	271	4-HyP	EPG	207	4-HyP	SPG	364
4-HyP	EPG	278	4-HyP	APG	210	4-HyP	EPG	370
4-HyP	SPG	281	4-HyP	TPG	216	HyP	PQG	372
4-HyP	APG	287	4-HyP	APG	234	4-HyP	PPG	382
4-HyP	LPG	296	4-HyP	PPG	261	4-HyP	PPG	385
4-HyP	RPG	302	4-HyP	FPG	264	4-HyP	SPG	391
4-HyP	PPG	305	4-HyP	APG	267	HyP	PAG	399
3-HyP	PPG	322	4-HyP	NPG	279	4-HyP	IPG	403
4-HyP	PPG	323	HyP	PAG	284	4-HyP	APG	406
HyP	PTG	325	4-HyP	LPG	294	4-HyP	PPG	415
HyP	PTG	328	3-HyP	PPG	302	4-HyP	IPG	424
3-HyP	PPG	331	4-HyP	PPG	303	4-HyP	EPG	433
4-HyP	PPG	332	4-HyP	NPG	306	4-HyP	SPG	454
4-HyP	FPG	335	HyK	AKG	315	4-HyP	IPG	457
HyK	AKG	341	4-HyP	LPG	321	4-HyP	SPG	469
G-HyK	AKG	341	4-HyP	APG	327	4-HyP	EPG	472
GG-HyK	AKG	341	4-HyP	LPG	330	4-HyP	LPG	478
4-HyP	EPG	362	4-HyP	IPG	336	4-HyP	IPG	499
3-HyP	PPG	364	HyP	PAG	338	HyK	EKG	502
4-HyP	PPG	365	4-HyP	EPG	354	4-HyP	PPG	505
HyP	PAG	367	4-HyP	EPG	369	4-HyP	GPG	511
HyP	PAG	373	4-HyP	PPG	378	4-HyP	EPG	523
4-HyP	NPG	377	4-HyP	SPG	390	4-HyP	TPG	529
4-HyP	QPG	383	HyP	PAG	398	4-HyP	GPG	532
HyK	AKG	386	3-HyP	PPG	401	4-HyP	MPG	538
4-HyP	APG	392	4-HyP	PPG	402	4-HyP	SPG	541
4-HyP	APG	398	4-HyP	SPG	408	4-HyP	GPG	544
4-HyP	FPG	401	4-HyP	LPG	414	4-HyP	PPG	553
HyP	PSG	406	4-HyP	PPG	426	4-HyP	QPG	574
HyP	PQG	409	4-HyP	RPG	447	4-HyP	FPG	580
HyP	PSG	412	4-HyP	EPG	450	HyP	PKG	582
3-HyP	PPG	415	HyP	PRG	455	HyK	PKG	583
4-HyP	PPG	416	4-HyP	LPG	459	4-HyP	APG	589
HyP	PKG	418	4-HyP	SPG	462	4-HyP	GPG	598
HyK	PKG	419	HyP	PVG	473	4-HyP	GPG	601
4-HyP	EPG	425	4-HyP	LPG	477	4-HyP	LPG	604
4-HyP	APG	428	4-HyP	RPG	483	4-HyP	APG	667
HyK	AKG	437	HyP	PAG	488	4-HyP	APG	670
4-HyP	EPG	440	4-HyP	FPG	501	HyK	GKG	673
4-HyP	PPG	449	4-HyP	DPG	510	4-HyP	APG	679
4-HyP	EPG	464	4-HyP	KPG	513	4-HyP	PPG	685
HyP	PSG	466	4-HyP	HPG	519	4-HyP	IPG	691
4-HyP	LPG	470	4-HyP	APG	528	4-HyP	PPG	700
4-HyP	PPG	473	HyP	PDG	530	4-HyP	PPG	712
4-HyP	GPG	479	4-HyP	PPG	540	4-HyP	PPG	715
4-HyP	FPG	485	4-HyP	PPG	558	4-HyP	SPG	721
HyK	PKG	494	4-HyP	LPG	564	4-HyP	MPG	727
HyP	PSG	496	4-HyP	KPG	576	4-HyP	GPG	733
4-HyP	APG	503	4-HyP	LPG	582	4-HyP	SPG	736
HyP	PAG	505	4-HyP	LPG	588	HyK	EKG	742
HyP	PKG	508	HyP	PAG	590	4-HyP	EPG	745
HyK	PKG	509	4-HyP	TPG	600	4-HyP	VPG	754
G-HyK	PKG	509	HyP	PSG	608	HyP	PAG	762
4-HyP	RPG	518	4-HyP	APG	621	HyP	PIG	765
4-HyP	LPG	524	4-HyP	APG	636	4-HyP	PPG	769
HyK	AKG	527	4-HyP	LPG	648	4-HyP	QPG	775
4-HyP	SPG	533	4-HyP	IPG	657	HyK	DKG	778
4-HyP	SPG	536	HyK	EKG	663	4-HyP	SPG	784
3-HyP	PPG	544	4-HyP	IPG	684	4-HyP	LPG	787
4-HyP	PPG	545	4-HyP	APG	690	4-HyP	GPG	796
4-HyP	RPG	554	HyP	PSG	707	4-HyP	PPG	805
HyP	PAG	556	4-HyP	SGP	717	4-HyP	FPG	811
4-HyP	FPG	572	HyP	PAG	725	4-HyP	APG	814
HyK	PKG	575	4-HyP	QPG	741	4-HyP	EPG	820
4-HyP	EPG	581	HyK	AKG	744	HyK	AKG	823
4-HyP	LPG	590	HyK	EKG	747	4-HyP	APG	829
4-HyP	PPG	593	HyK	TKG	750	HyK	EKG	832
4-HyP	APG	611	HyK	PKG	753	4-HyP	PPG	838
4-HyP	SPG	629	4-HyP	PPG	777	4-HyP	PPG	853
4-HyP	LPG	635	4-HyP	PPG	789	HyK	VKG	859
4-HyP	PPG	641	4-HyP	FPG	795	4-HyP	SPG	865
4-HyP	KPG	647	3-HyP	PPG	803	4-HyP	GPG	868
4-HyP	VPG	653	4-HyP	PPG	804	4-HyP	FPG	874
4-HyP	APG	659	HyP	PSG	806	4-HyP	LPG	880
4-HyP	FPG	671	4-HyP	PPG	813	3-HyP	PPG	882
4-HyP	PPG	680	4-HyP	PPG	816	4-HyP	PPG	883
HyP	PRG	685	4-HyP	PPG	846	4-HyP	NPG	889
4-HyP	APG	704	4-HyP	EPG	858	3-HyP	PPG	891
4-HyP	APG	707	4-HyP	APG	864	4-HyP	PPG	892
4-HyP	APG	713	4-HyP	APG	876	4-HyP	APG	898
4-HyP	MPG	719	4-HyP	LPG	882	4-HyP	PPG	904
4-HyP	LPG	728	4-HyP	LPG	891	HyP	PAG	906
HyK	PKG	731	4-HyP	EPG	900	4-HyP	SPG	913
G-HyK	PKG	731	4-HyP	PPG	909	4-HyP	NPG	916
3-HyP	PPG	760	4-HyP	PPG	915	4-HyP	QPG	928
4-HyP	PPG	761	4-HyP	SPG	921	4-HyP	PPG	934
4-HyP	APG	767	4-HyP	APG	927	4-HyP	PPG	940
4-HyP	PPG	779	4-HyP	NPG	936	4-HyP	SPG	943
4-HyP	APG	788	3-HyP	PPG	941	4-HyP	PPG	961
4-HyP	PPG	797	4-HyP	PPG	942	4-HyP	MPG	964
4-HyP	PPG	806	4-HyP	QPG	948	4-HyP	SPG	970
4-HyP	QPG	812	HyK	HKG	951	HyK	IKG	976
4-HyP	EPG	818	4-HyP	YPG	957	4-HyP	KPG	982
HyK	VKG	824	4-HyP	APG	969	4-HyP	PPG	994
4-HyP	PPG	830	4-HyP	EPG	987	4-HyP	LPG	1000
4-HyP	PPG	839	HyP	PAG	989	4-HyP	QPG	1003
4-HyP	APG	848	HyP	PQG	1007	4-HyP	EPG	1009
3-HyP	PPG	859	HyK	DKG	1014	4-HyP	NPG	1015
4-HyP	PPG	860	4-HyP	EPG	1017	4-HyP	QPG	1021
4-HyP	FPG	866	HyK	DKG	1020	4-HyP	SPG	1027
3-HyP	PPG	874	4-HyP	LPG	1026	4-HyP	SPG	1039
4-HyP	PPG	875	HyK	LKG	1029	4-HyP	APG	1042
4-HyP	PPG	884	4-HyP	LPG	1038	4-HyP	APG	1045
4-HyP	PPG	887	4-HyP	APG	1050	4-HyP	HPG	1048
4-HyP	RPG	908	HyP	PAG	1061	4-HyP	PPG	1051
4-HyP	PPG	914	HyP	PSG	1064	HyP	PSG	1071
4-HyP	PPG	917	4-HyP	QPG	1077	4-HyP	APG	1075
4-HyP	SPG	926				HyP	PAG	1077
HyP	PAG	931				4-HyP	APG	1084
4-HyP	SPG	935				HyP	PQG	1086
4-HyP	TPG	938				HyK	DKG	1093
4-HyP	LPG	953				4-HyP	FPG	1111
4-HyP	FPG	962				4-HyP	NPG	1114
4-HyP	LPG	965				4-HyP	PPG	1117
HyP	PSG	967				4-HyP	SPG	1120
4-HyP	EPG	971				4-HyP	SPG	1132
3-HyP	PPG	985				4-HyP	PPG	1147
4-HyP	PPG	986				4-HyP	HPG	1156
4-HyP	PPG	992				4-HyP	PPG	1162
HyP	PMG	988				HyP	PRG	1164
4-HyP	PPG	998						
4-HyP	SPG	1007						
4-HyP	SPG	1013						
4-HyP	APG	1019						
HyK	AKG	1022						
3-HyP	PPG	1033						
4-HyP	PPG	1034						
4-HyP	APG	1037						
4-HyP	APG	1040						
4-HyP	APG	1043						
HyP	PAG	1063						
HyP	PAG	1075						
HyP	PRG	1081						
HyK	DKG	1085						
3-HyP	PPG	1108						
4-HyP	PPG	1109						
4-HyP	SPG	1112						
4-HyP	SPG	1115						
4-HyP	PPG	1133						
4-HyP	SPG	1139						
4-HyP	LPG	1148						
3-HyP	PPG	1153						
4-HyP	PPG	1154						

On the wild-type mice skin COL1A1 chain, a total of 160 site-specific PTMs were detected. A total of 106 4-hydroxyproline sites, 12 3-hydroxyproline sites, 22 hydroxyproline sites, 14 5-hydroxylysine sites, four galactosyl-hydroxylysine sites, and two glucosylgalactosyl-hydroxylysine sites on COL1A1 were identified. On COL1A2 extracted from wild-type mice skin, a total of 124 PTM sites were identified in this analysis. A total of 89 4-hydroxyproline sites, four 3-hydroxyproline sites, 17 hydroxyproline sites, and 14 5-hydroxylysine sites were detected on COL1A2. In this analysis, no O-glycosylation site was detected on COL1A2 extracted from the skin of wild-type mice.

Except for these two chains of collagen I, site-specific PTMs on collagen III, which form a homotrimer of COL3A1 chains, were also identified. A total of 137 PTM sites on wild-type mice skin COL3A1 were identified. COL3A1 was found to have 112 4-hydroxyproline sites, two 3-hydroxyproline sites, 10 hydroxyproline sites, 12 5-hydroxylysine sites, and one glucosyl-galactosyl-hydroxylysine site. In this analysis, a total of 421 site-specific collagen PTMs were identified by MS2-level peptide-spectrum match-based validation on COL1A1, COL1A2, and COL3A1 chains extracted from wild-type mice skin. COL1A1 was found to be the most modified and COL1A2 to be the least modified among the three fibrillar collagen chains.

### 3.2 Mass spectrometry-based validation of the -Xaa-Pro-Gly- motif catalyzed by prolyl 4-hydroxylases

**FIGURE 1:**
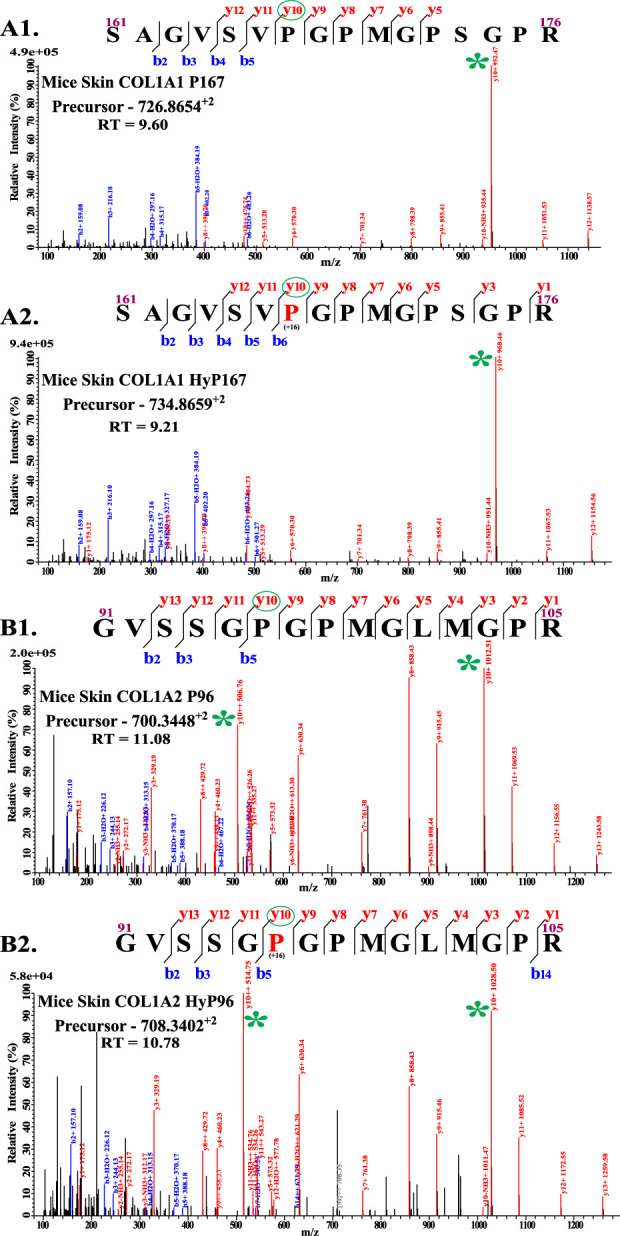
Validation of C-P4h specific motif (-Xaa-Pro-Gly-). **(A1)** shows the unmodified peptide from COL1A1 with residues numbered 161–176. **(A2)** shows the presence of hydroxyproline on P167 and the presence of hydroxyproline on the -Xaa-Pro-Gly- motif. Similarly, **(B1)** shows an unmodified peptide from COL1A2 (91–105), and **(B2)** shows the presence of hydroxyproline on P96 on the “Yaa” position of the -Xaa-Yaa-Gly- motif.

There are -Gly-Xaa-Yaa- repeats present in the helical region of collagen 1. During the proteomics analyses, proline present on the Yaa position of -Gly-Xaa-Yaa- or -Gly-Xaa-Yaa-Gly- motif was considered to be 4-hydroxylated. However, previous studies show that proline present in the -Xaa-Pro-Gly- motif is catalyzed by prolyl 4-hydroxylases C-P4Hs (Pihlajaniemi et al., 1991; Myllyharju and Kivirikko, 1999; Pekkala et al., 2003; Koski et al., 2009; Perdivara et al., 2013). In this regard, the MS analysis shows an interesting finding (Figure 1). This study shows that glycine before -Xaa-Pro- is not required for the catalysis of hydroxylation on proline by C-P4Hs (Figures 1A2, B2); rather, glycine is found after the -Xaa-Pro- position in all identified 4-hydroxyproline sites (Table 1). The presence of hydroxylation on COL1A1 P167 and COL1A2 P96 depicts that glycine is not required before -Xaa-Pro- (Xaa can be any naturally occurring amino acid) for motif recognition by C-P4Hs. The COL1A1 P167 does not fit in the “-Gly-Xaa-Yaa-” or “-Gly-Xaa-Yaa-Gly-” motifs because serine (S) and valine (V) are present on 165 and 166 sites, respectively (Figure 1A2). Glycine is not present on the COL1A1 165 site, but it is present on the 168 sites. This means that site COL1A1 P167 only fits in the “-Xaa-Yaa-Gly-” motif. Similarly, in the case of COL1A2 P96, serine is present before -Xaa-Pro- (Figure 1B2), and P96 fits only in the “-Xaa-Yaa-Gly-” motif. Moreover, every other proline modification site shown in Table 1 follows the -Xaa-Yaa-Gly- motif. Collagen contains -Xaa-Yaa-Gly- repeats in the helical region of collagen 1. Repetition of these -Xaa-Yaa-Gly motifs led to the confusion of considering either -Gly-Xaa-Yaa- or -Gly-Xaa-Yaa-Gly- to be the preferred motif of C-P4H activity. Previously, several groups have shown that -Xaa-Yaa-Gly- is the C-P4H-specific motif on collagen (Risteli and Kivirikko, 1974; Pihlajaniemi et al., 1991; Myllyharju and Kivirikko, 1999; Pekkala et al., 2003; Sipila et al., 2018; Tolonen et al., 2022). This study provides proteomics-based evidence (Figure 1) for the “-Xaa-Yaa-Gly-” motif validation.

Table 1 shows that all 4-hydroxyproline sites have a -Xaa-Pro-Gly motif. Of 106 4-hydroxyproline sites on wild-type mice skin COL1A1, 17 4-hydroxyproline sites were detected on -Ala-Pro-Gly- motif, 10 4-hydroxyproline sites were detected on -Glu-Pro-Gly- motif, and seven 4-hydroxyproline sites are detected on -Phe-Pro-Gly- motif. In this analysis, the most common motif for 4-hydroxyproline is the -Pro-Pro-Gly- motif, and 35 4-hydroxyproline sites on this motif were identified. A total of 12 4-hydroxyproline sites on the -Leu-Pro-Gly- motif, five 4-hydroxyproline sites on the -Arg-Pro-Gly- motif, 11 4-hydroxyproline sites on the -Ser-Pro-Gly- motif, two 4-hydroxyproline sites on the -Gln-Pro-Gly- motif and -Val-Pro-Gly- motifs, and one site each on the -Gly-Pro-Gly- motif, -Lys-Pro-Gly-, -Met-Pro-Gly-, -Asn-Pro-Gly- motif, and -Thr-Pro-Gly- motif were detected. Similarly to wild-type COL1A1, 4-hydroxyproline sites were present in -Xaa-Pro-Gly- motifs in COL1A2 and COL3A1 chains.

### 3.3 Identification of site-specificity of C-P4Hs in fibrillar collagens

C-P4Hs modify proline residues present on the -Xaa-Pro-Gly- motif in collagens. However, collagen-modifying enzymes are known to have site-specificity (Morello et al., 2006; Pokidysheva et al., 2013; 2014; Hudson et al., 2015; Terajima et al., 2016; Tonelli et al., 2020; Ishikawa et al., 2022). In this study, 4-HyP occupancy percentage quantitation was performed to determine site-specificity of P4HA1 and P4HA2 enzymes on COL1A1, COL1A2, and COL3A1 extracted from skin of wild-type and C-P4Hs mutant (*P4ha1* ^*+/−*^; *P4ha2−/−*, *P4ha1* ^*+/+*^; *P4ha2−/−*, *P4ha1* ^*+/−*^; *P4ha2* ^*+/−*^, *P4ha1* ^*+/+*^; *P4ha2* ^*+/−*^) mice.

#### 3.3.1 Identification of site-specificity of P4HA1 in fibrillar collagens

P4HA1 is the predominant C-P4H form in mice skin, constituting ∼83% of all C-P4Hs (Salo et al., 2024). P4HA1 is essential for the survival of a mouse. A complete deletion (*P4ha1−/−*) of *P4ha1* is embryonically lethal in mice (Sipila et al., 2018; Tolonen et al., 2022). P4HA1 null mice are not viable. This means that P4HA1 activity on collagen is highly important for the development of the mouse body. P4HA1 is a prolyl 4-hydroxylase enzyme; this means that some 4-hydroxyproline sites are pivotal for mouse development, and without 4-hydroxylation on these proline sites, mice cannot survive. Interestingly, *P4ha1* ± mutant mice are viable. Prolyl 4-hydroxylation activity is still retained in mice upon partial deletion of *P4ha1* (*P4ha1* ^*+/−*^). Surprisingly, a total of 23 sites on mice skin COL1A1, COL1A2, and COL3A1 chains were found to be fully (≥99%) 4-hydroxylated in the wild type as well as in *P4ha1* and *P4ha2* mutants (*P4ha1* ^*+/−*^; *P4ha2−/−*, *P4ha1* ^*+/+*^; *P4ha2−/−*, *P4ha1* ^*+/−*^; *P4ha2* ^*+/−*^, *P4ha1* ^*+/+*^; *P4ha2* ^*+/−*^) (Table 2).

**TABLE 2:** P4HA1-specific 4-hydroxyproline sites with occupancy (%) in mouse skin fibrillar collagen chains.

Site	Motif	WT	*P4ha1* ^*+/+*^; *P4ha2* ^*+/−*^	*P4ha1* ^*+/−*^; *P4ha2* ^*+/−*^	*P4ha1* ^*+/+*^; *P4ha2−/−*	*P4ha1* ^*+/−*^; *P4ha2−/−*
COL1A1 P236	PPG	100	100	100	100	100
COL1A1 P245	LPG	100	100	100	100	100
COL1A1 P305	PPG	100	100	100	100	100
COL1A1 P533	SPG	100	100	100	100	100
COL1A1 P875	PPG	100	100	100	100	100
COL1A1 P629	SPG	100	100	100	100	100
COL1A1 P728	LPG	100	100	100	100	100
COL1A1 P860	PPG	99.82 ± 0.03	99.71 ± 0.15	99.03 ± 0.29	99.89 ± 0.19	99.93 ± 0.04
COL1A1 P866	FPG	99.96 ± 0.05	99.93 ± 0.07	99.53 ± 0.13	99.89 ± 0.20	99.96 ± 0.04
COL1A2 P174	FPG	100	100	100	100	100
COL1A2 P321	LPG	100	100	100	100	100
COL1A2 P327	APG	100	100	100	100	100
COL1A2 P540	PPG	100	100	100	100	100
COL1A2 P891	LPG	100	100	100	100	100
COL1A2 P957	YPG	100	100	100	100	100
COL1A2 P426	PPG	98.98 ± 1.22	99.79 ± 0.18	99.68 ± 0.46	99.88 ± 0.13	99.40 ± 0.29
COL3A1 P454	SPG	100	100	100	100	100
COL3A1 P667	APG	100	100	100	100	100
COL3A1 P865	SPG	100	100	100	100	100
COL3A1 P868	GPG	100	100	100	100	100
COL3A1 P874	FPG	100	100	100	100	100
COL3A1 P532	GPG	100	100	100	99.85 ± 0.09	99.42 ± 0.11
COL3A1 P580	FPG	100	100	100	100	100

Interestingly, neither the occupancy percentages of these specific 4-HyP sites nor the partial deletion of *P4ha1* (*P4ha1* ^*+/−*^) decreased upon the complete deletion of *P4ha2* (*P4ha2−/−*). These findings hint that P4HA2 does not catalyze 4-hydroxylation on these 23 sites (Table 2). On the other hand, P4HA3 constitutes only ∼2% of C-P4Hs in wild-type mice skin (Salo et al., 2024). Because P4HA1 is the most abundant (∼83% abundance) C-P4H isoform in the skin, even after partial deletion, it is likely to be present in higher amounts than P4HA2 and P4HA3. This indicates that even after partial deletion of gene *P4ha1* (*P4ha1* ^*+/−*^), the P4HA1 enzyme retains the ability to fully catalyze the 4-hydroxylation on these 23 proline sites. These sites have specificity for P4HA1 in mice skin, as the 4-HyP occupancy percentages on these sites are not affected by the absence/reduced abundance of other C-P4Hs (Table 2).

#### 3.3.2 Identification of site-specificity of P4HA2 in fibrillar collagens

**FIGURE 2:**
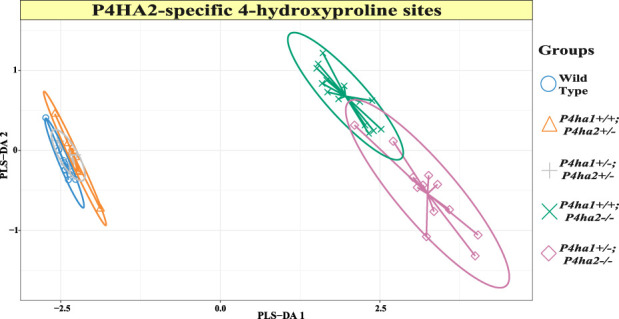
Expression of 4-hydroxyproline occupancy percentages on P4HA2-specific sites. The sPLS-DA plot of seven P4HA2-specific 4-hydroxyproline site occupancy percentages shows that the 4-hydroxyproline expression is almost similar in wild-type, *P4ha1* ^*+/+*^; *P4ha2* ^*+/−*^, and *P4ha1* ^*+/−*^; *P4ha2* ± mice. However, the expression of occupancy percentages of these seven 4-hydroxyproline sites is different in mice with a complete deletion of *P4ha2* (*P4ha1* ^*+/+*^; *P4ha2−/−*, and *P4ha1* ^*+/−*^; *P4ha2−/−*).

**FIGURE 3:**
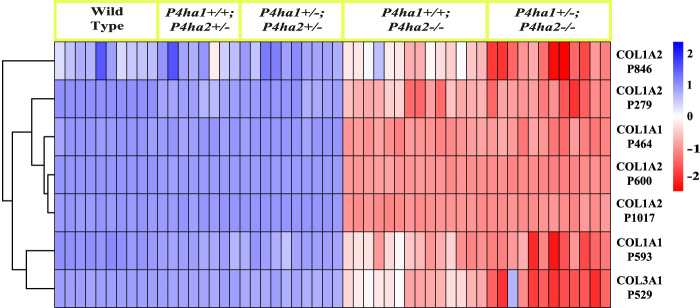
Occupancy percentages of P4HA2-specific 4-HyP sites. The heatmap shows that the 4-hydroxyproline occupancy percentages on seven fibrillar collagen sites are almost similar in wild-type, *P4ha1* ^*+/+*^; *P4ha2* ^*+/−*^, and *P4ha1* ^*+/−*^; *P4ha2* ± mice, but occupancy is significantly decreased in mice with a complete deletion of *P4ha2* (*P4ha1* ^*+/+*^; *P4ha2−/−*, and *P4ha1* ^*+/−*^; *P4ha2−/−*).

**FIGURE 4:**
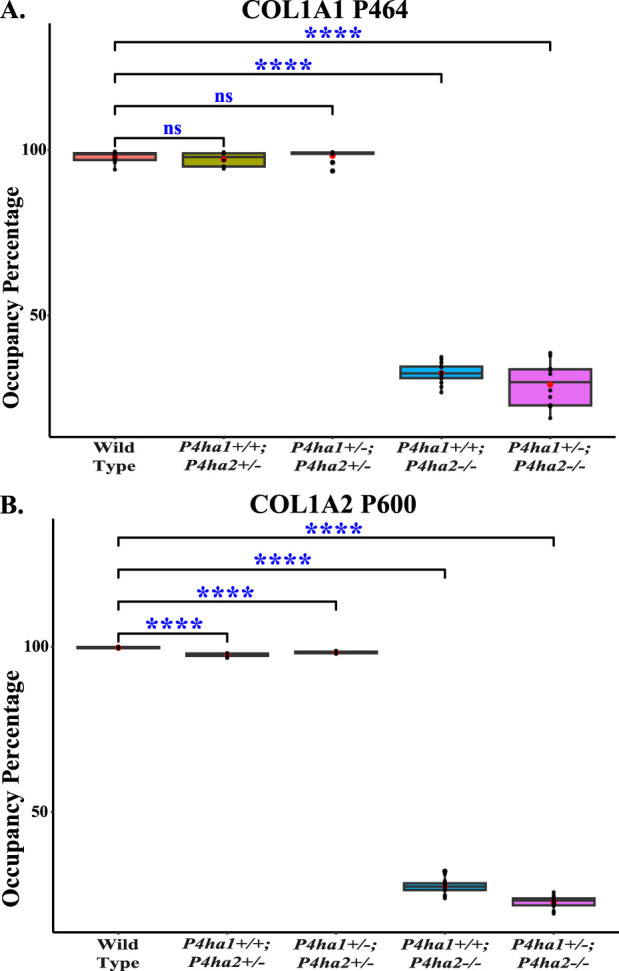
Occupancy percentages of COL1A1 P464 and COL1A2 P600. On the P4HA2-specific sites P464 **(A)** and P600 **(B)**, the 4-hydroxyproline occupancy is almost similar in wild-type, *P4ha1* ^*+/+*^; *P4ha2* ^*+/−*^, and *P4ha1* ^*+/−*^; *P4ha2* ± mice, but occupancy is significantly decreased in mice with a complete deletion of *P4ha2* (*P4ha1* ^*+/+*^; *P4ha2−/−*, and *P4ha1* ^*+/−*^; *P4ha2−/−*). In the figure, (ns) represents a p-value >0.05, and (****) represents a p-value <0.0001. The p-value is calculated using the Student’s t-test, conducted on wild-type and respective prolyl 4-hydroxylase mutant mice.

P4HA2 is the second-most abundant isoform of the C-P4H family, and it constitutes ∼15% of all C-P4Hs in mice skin. Interestingly, this study reveals P4HA2-specific 4-HyP sites that are not compensated by predominant P4HA1 in the absence of P4HA2 in mice skin. Interestingly, this study shows that partial deletion (*P4ha2* ^*+/−*^ *)* and complete deletion of *P4ha2* (*P4ha2−/−)* differently affect the 4-hydroxyproline level in seven fibrillar collagen sites (Figure 2). In this analysis, seven 4-HyP sites of mice skin COL1A1, COL1A2, and COL3A1 chains were found to have more than 95% occupancy in wild type, *P4ha1* ^*+/+*^; *P4ha2* ^*+/−*^, and *P4ha1* ^*+/−*^; *P4ha2* ± mice. In mice with a single allele deletion of *P4ha2* (*P4ha2* ^*+/−*^ *),* the 4-hydroxyproline occupancy percentages on these seven sites (Table 3; Figure 3) were similar to wild-type mice. However, the 4-hydroxyproline occupancy percentages on these seven sites were significantly decreased (Figure 3; Table 3) in mice with a complete deletion of *P4ha2* (*P4ha1* ^*+/+*^; *P4ha2−/−* and *P4ha1* ^*+/−*^; *P4ha2−/−*). These findings indicate that P4HA2 is required to fully modify these sites, and other C-P4Hs were not able to sufficiently modify these seven sites site up to the level of the wild-type mice group upon complete deletion (Figures 2–4; Table 3).

**TABLE 3:** P4HA2-specific 4-hydroxyproline sites with occupancy (%) in mouse skin. In the table, (*) represents a p-value <0.05 calculated using Student’s t-test, conducted on wild-type and prolyl 4-hydroxylase mutant mice.

Site	Motif	WT	*P4ha1* ^*+/+*^; *P4ha2* ^*+/−*^	*P4ha1* ^*+/−*^; *P4ha2* ^*+/−*^	*P4ha1* ^*+/+*^; *P4ha2−/−*	*P4ha1* ^*+/−*^; *P4ha2−/−*
COL1A1 P593	PPG	99.45 ± 0.31	98.19 ± 0.47	98.13 ± 0.89	87.16 ± 2.59*	81.71 ± 3.09*
COL1A1 P464	EPG	97.87 ± 1.63	97.17 ± 2.02	98.26 ± 1.77	32.58 ± 2.95*	29.13 ± 6.38*
COL1A2 P279	NPG	99.97 ± 0.05	97.42 ± 0.96	98.76 ± 1.05	78.04 ± 3.86*	73.54 ± 3.61*
COL1A2 P600	TPG	99.70 ± 0.18	97.51 ± 0.48	98.27 ± 0.28	27.56 ± 2.25*	22.85 ± 1.80*
COL1A2 P846	PPG	97.24 ± 0.98	97.48 ± 1.43	97.94 ± 0.58	94.50 ± 1.05*	90.77 ± 1.48*
COL1A2 P1017	EPG	98.33 ± 1.30	98.22 ± 1.37	99.41 ± 0.66	3.80 ± 2.20*	4.10 ± 3.00*
COL3A1 P529	TPG	99.95 ± 0.03	99.83 ± 0.09	99.62 ± 0.14	94.99 ± 1.09*	91.38 ± 2.53*

### 3.4 Analysis of the effects of *P4ha1* and *P4ha2* deletion on other site-specific PTMs of mouse skin collagen I

In order to delineate the effect of *P4ha1* and *P4ha2* deletion on collagen PTMs other than 4-hydroxyproline, the site-specific quantitation of 3-hydroxyproline, 5-hydroxylysine, and O-glycosylation sites was performed on COL1A1 and COL1A2 chains extracted from the skin of wild-type mice and C-P4H mutant (*P4ha1* ^*+/−*^; *P4ha2−/−*, *P4ha1* ^*+/+*^; *P4ha2−/−*, *P4ha1* ^*+/−*^; *P4ha2* ^*+/−*^, *P4ha1* ^*+/+*^; *P4ha2* ^*+/−*^) mice.

#### 3.4.1 Analysis of the effect of *P4ha1* and *P4ha2* deletion on the site-specific 3-hydroxylation on mouse skin collagen I

The effects of *P4ha1* and *P4ha2* deletion on the occupancy percentages of prolyl 3-hydroxylation sites on COL1A1 and COL1A2 were analyzed in this study. P3H1 is a prominent prolyl 3-hydroxylase. P3H1 catalyzes the site-specific prolyl 3-hydroxylation on collagen I (COL1A1 and COL1A2) chains (Cabral et al., 2007; Vranka et al., 2010; Pokidysheva et al., 2013). In COL1A1, 3-HyP sites, that is, P1153 (it is site P986 in cleaved COL1A1) and P874 (P707), are reported to be 3-hydroxylated by P3H1 (Pokidysheva et al., 2013) and in COL1A2, P803 (P707) is modified by P3H1 in mice bone (Pokidysheva et al., 2013). In this study, quantitation of 3-hydroxyproline occupancy percentage on these three classical 3-HyP sites of collagen 1 was performed. On site COL1A1 P1153, a 94.65% occupancy percentage was detected in wild-type mice skin, which is in accordance with the previous findings by Pokidysheva et al. (2013). Additionally, on COL1A1 P874 and COL1A2 P803, respectively, 5.7% and 13.55% occupancy percentages were detected in wild-type mice skin. However, these COL1A1 P874 and COL1A2 P803 sites were not previously detected in mice skin by Pokidysheva et al. (2013).

**FIGURE 5:**
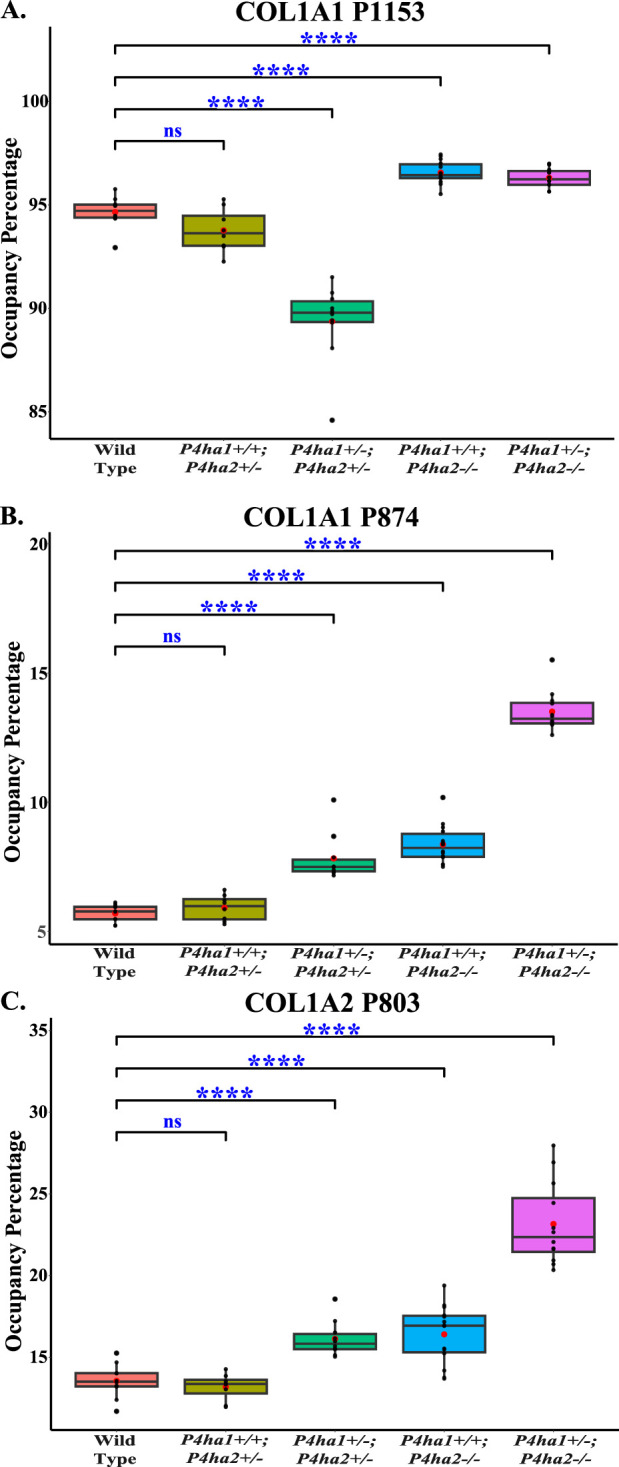
Altered occupancy percentages of P3H1-specific 3-hydroxyproline sites of collagen I upon *P4ha1* and *P4ha2* deletion. **(A)** The 3-hydroxyproline occupancy percentage on COL1A1 P1153 (P986) is decreased compared to wild type upon partial deletion of *P4ha1* and *P4ha2* but is increased upon complete deletion of *P4ha2*. On the other hand, 3-hydroxyproline occupancy on **(B)** COL1A1 P874 (COL1A1 P707) and **(C)** COL1A1 P803 (P707) is elevated compared to wild-type mice upon partial deletion of *P4ha1* and/or complete deletion of *P4ha2*. In the figure, (ns) represents a p-value >0.05, and (****) represents a p-value <0.0001. The p-value is calculated using the Student’s t-test, conducted on wild-type and respective prolyl 4-hydroxylase mutant mice.

Interestingly, this study reveals that partial deletion of *P4ha1* (*P4ha1* ^*+/−*^) and complete deletion of *P4ha2* (*P4ha2−/−*) significantly increases the 3-hydroxyproline occupancy percentages on COL1A1 P874 and COL1A2 P803 sites compared to wild-type mice (Figure 5; Table 4). On the COL1A1 P1153 site, a significant decrease in 3-hydroxyproline occupancy percentage in *P4ha1* ^*+/−*^; *P4ha2* ± mice was detected compared to wild-type mice (Figure 5; Table 4). However, the 3-HyP occupancy of P1153 was significantly increased in complete *P4ha2* (*P4ha2−/−*) deleted mutant mice similar to COL1A1 P874 and COL1A2 P803 sites (Figure 5; Table 4). This indicates that complete deletion of *P4ha2* (*P4ha2−/−*) enhances the prolyl 3-hydroxylation on specific sites on collagen 1.

**TABLE 4:** Quantitation of 3-hydroxyproline occupancy (%) on P3H1-specific sites in collagen I. In the table, (*) represents a p-value <0.05 calculated using Student’s t-test, conducted on wild-type and respective prolyl 4-hydroxylase mutant mice.

Site	WT	*P4ha1* ^*+/+*^; *P4ha2* ^*+/−*^	*P4ha1* ^*+/−*^; *P4ha2* ^*+/−*^	*P4ha1* ^*+/+*^; *P4ha2−/−*	*P4ha1* ^*+/−*^; *P4ha2−/−*
COL1A1 P874	5.70 ± 0.31	5.92 ± 0.46^ns^	7.83 ± 0.86*	8.37 ± 0.72*	13.51 ± 0.74*
COL1A1 P1153	94.65 ± 0.72	93.77 ± 0.98^ns^	89.37 ± 1.81*	96.56 ± 0.53*	96.30 ± 0.44*
COL1A2 P803	13.55 ± 0.98	13.18 ± 0.77^ns^	16.13 ± 1.02*	16.40 ± 1.71*	23.16 ± 2.42*

#### 3.4.2 Analysis of the effects of *P4ha1* and *P4ha2* deletion on site-specific lysyl 5-hydroxylation on mouse skin collagen I

**FIGURE 6:**
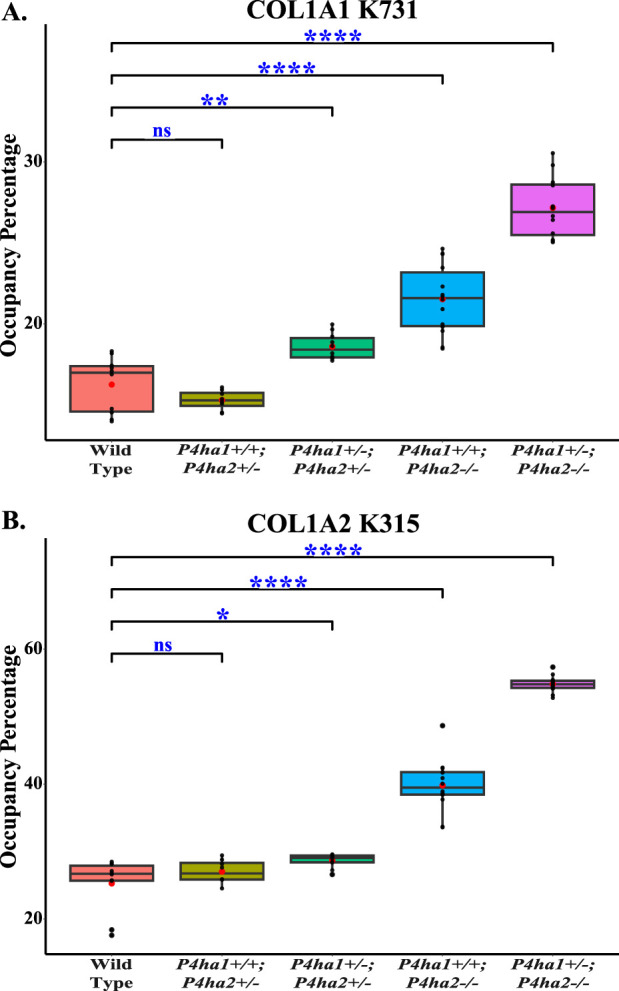
Altered occupancy percentages of two non-cross-linking helical hydroxylysine sites upon deletion of *P4ha1* and *P4ha2*. Occupancy percentages of collagen I helical cross-linking sites in wild-type and C-P4h mutant mice. 5-hydroxylysine occupancy percentages on COL1A1 K731 **(A)** and COL1A2 K315 **(B)** are increased upon partial deletion of *P4ha1* and/or complete deletion of *P4ha2*. In the figure, (ns) represents a p-value >0.05, (*) represents a p-value <0.05, (**) represents a p-value <0.01, and (****) represents a p-value <0.0001. The p-value is calculated using Student’s t-test, conducted on wild-type and respective prolyl 4-hydroxylase mutant mice.

In collagens, lysines are also heavily modified. Lysine sites are vital for collagen cross-linking in the extracellular matrix. In this study, quantitation of the occupancy percentages of hydroxylysine and O-glycosylation on helical collagen cross-linking lysine sites and helical collagen non-cross-linking lysine sites was performed. It was found that COL1A1 N-terminal helical cross-linking site K254 (generally known as K87) was fully (∼99%) (Table 5) modified with glucosylgalactosyl-hydroxylysine (GG-HyK) in wild-type mice and in C-P4H mutant (*P4ha1* ^*+/−*^; *P4ha2−/−*, *P4ha1* ^*+/+*^; *P4ha2−/−*, *P4ha1* ^*+/−*^; *P4ha2* ^*+/−*^, *P4ha1* ^*+/+*^; *P4ha2* ^*+/−*^) mice. Interestingly, it was detected that partial deletion of *P4ha1* and complete deletion of *P4ha2* significantly increased the hydroxylysine level in COL1A2 N-terminal helical cross-linking site K183 (Figure 6; Table 5). Similarly, an elevation in hydroxylysine occupancy percentage in non-cross-linking COL1A1 K731 and COL1A2 K315 sites was also detected. Moreover, the occupancy percentages of COL1A1 K731 galactosyl-hydroxylysine (G-HyK) were found to be elevated upon partial deletion of *P4ha1* and complete deletion of *P4ha2* (Table 5). These findings indicate that prolyl 4-hydroxylases play a crucial role in the hydroxylation of helical cross-linking lysine sites and helical non-cross-linking lysine sites in collagen 1 (Figure 6; Table 5).

**TABLE 5:** Occupancy (%) levels of lysyl hydroxylation and O-glycosylation on collagen I helical cross-linking and non-cross-linking sites. In the table, (*) represents a p-value <0.05, and (ns) represents a p-value>0.05 calculated using Student’s t-test, conducted on wild-type and respective prolyl 4-hydroxylase mutant mice.

Site	WT	*P4ha1* ^*+/+*^; *P4ha2* ^*+/−*^	*P4ha1* ^*+/−*^; *P4ha2* ^*+/−*^	*P4ha1* ^*+/+*^; *P4ha2−/−*	*P4ha1* ^*+/−*^; *P4ha2−/−*
Helical XL sites					
COL1A1 GG-HyK254	99.62 ± 0.22	99.68 ± 0.19	99.88 ± 0.08	99.86 ± 0.08	99.77 ± 0.18
COL1A2 HyK183	89.78 ± 0.96	91.25 ± 1.41*	95.01 ± 1.84*	97.60 ± 2.13*	96.97 ± 3.42*
Helical non-XL sites					
COL1A2 HyK315	25.28 ± 3.76	27.01 ± 1.61^ns^	28.66 ± 0.97*	39.81 ± 3.65*	54.82 ± 1.18*
COL1A1 HyK731	16.25 ± 1.63	15.29 ± 0.56^ns^	18.60 ± 0.76*	21.52 ± 2.03*	27.17 ± 1.80*
COL1A1 G-HyK731	0.70 ± 0.14	0.69 ± 0.06^ns^	1.19 ± 0.13*	1.79 ± 0.29*	3.58 ± 0.41*

## 4 Discussion

In wild-type mice skin, P4HA1 is the most abundant isoform among all three C-P4Hs. P4HA1 constitutes ∼83% and P4HA2 constitutes ∼15% of total C-P4Hs in wild-type mice skin (Salo et al., 2024). P4HA1 has 5-fold more abundance than the second most abundant C-P4H isoform, that is, P4HA2. Complete deletion of *P4ha1* (*P4ha1−/−*) is embryonically lethal in mice (Sipila et al., 2018; Tolonen et al., 2022). However, mice with a partial deletion of *P4ha1* (*P4ha1* ^*+/−*^) are viable. This means that the P4HA1 enzyme encoded by only one allele (*P4ha1* ^*+/−*^) can also catalyze the modifications of specific collagen sites that are important for mouse survival. On the other hand, mice with a partial (*P4ha2* ^*+/−*^) or complete deletion of *P4ha2* (*P4ha2−/−*) are viable and do not have any visible phenotype (Sipila et al., 2018; Tolonen et al., 2022). This fact makes it interesting to decode the contribution of P4HA2 in mice skin collagen networks.

In this study, the effects of partial deletion of *P4ha1* (*P4ha1* ^*+/−*^), partial (*P4ha2* ^*+/−*^), and complete deletion of *P4ha2* (*P4ha2−/−*) on 4-hydroxyproline sites were evaluated in detail to delineate the site-specificity of P4HA1 and P4HA2 enzymes. Surprisingly, 4-hydroxyproline occupancy percentages on 23 sites were found to be around 100% (≥99%) in wild-type as well as mutant mice with partial *P4ha1* deletion, partial *P4ha2* deletion and complete *P4ha2* deletion (*P4ha1* ^*+/−*^; *P4ha2−/−*, *P4ha1* ^*+/+*^; *P4ha2−/−*, *P4ha1* ^*+/−*^; *P4ha2* ^*+/−*^, *P4ha1* ^*+/+*^; *P4ha2* ^*+/−*^) (Table 2). The occupancy percentages of 4-hydroxylation on these proline sites did not decrease upon partial deletion of *P4ha1* and *P4ha2*. Moreover, 4-hydroxyproline occupancy percentages on these 23 sites did not decrease even upon complete deletion of *P4ha2* (*P4ha2−/−*). Because P4HA1 has the highest (∼83%) abundance among C-P4Hs in wild-type mice skin (Sipila et al., 2018; Tolonen et al., 2022; Salo et al., 2024), it is plausible that the P4HA1 enzyme encoded by only one allele *P4ha1* (+/−) retains its prolyl 4-hydroxylation catalytic activity and can fully (∼100%) modify these 23 sites (Table 2). Except for these 23 sites, 4-hydroxyproline occupancy percentages on COL1A1 P230 (GRPGER), COL1A1 P671 (GFPGER), COL1A1 P719 (GMPGER), and COL1A2 P159 (GRPGER) were also found to be ∼100%, as previously detected by Sipila et al. (2018). Full occupancy of 4-hydroxyproline on these 27 sites indicates that these sites have a high significance in collagen triple helix formation, triple helix stability, and function of fibrillar collagens.

This study also identified P4HA2-specific sites in fibrillar collagen chains. The 4-hydroxyproline occupancy percentages on seven sites (Table 3) were found to be >95% in wild-type, *P4ha1* ^*+/+*^; *P4ha2* ^*+/−*^, and *P4ha1* ^*+/−*^; *P4ha2* ± mice. However, the occupancy percentages on these seven sites were significantly decreased in mice with a complete deletion of *P4ha2 (P4ha1* ^*+/−*^; *P4ha2−/−* and *P4ha1* ^*+/+*^; *P4ha2−/−*). Among these seven sites, three sites (COL1A1 P464, COL1A2 P600, and COL1A2 P1017) were found to have only <35% occupancy percentages in mice with a complete deletion of *P4ha2* compared to >95% occupancy percentages detected in wild-type and partial *P4ha1/P4ha2* deleted mice (Figures 3, 4; Table 3). The decreased occupancy percentages on these seven sites in the absence of P4HA2 indicate that these sites have specificity for P4HA2, and these sites cannot be sufficiently (>95%) modified by P4HA1 and P4HA3 enzymes. These P4HA2-specific sites have >95% 4-hydroxylation occupancy in wild type, so these sites might have some role in collagen biosynthesis and collagen interactions with extracellular proteins and cell-surface receptors.

Recently, Salo et al. (2024) reported that the complete deletion of *P4ha2* reduces the melting temperature of collagen and also affects collagen fibril assembly in the ECM. However, high levels of P4HA2 are associated with poor prognosis and progression of metastasis in many tissues of the human body (Aggarwal et al., 2022; Lu et al., 2022). Interestingly, it has even been reported that inhibition/absence of P4HA2 attenuates the metastasis progression and reduces the deposition of collagen in ECM (Lin et al., 2021; Shang et al., 2022). This indicates that although the basal levels of P4HA2 are required for proper collagen folding and collagen fibril assembly, elevated levels of P4HA2 can facilitate the progression of pathophysiological complications where collagen excessively gets deposited in the ECM. Therefore, P4HA2 can be a potential therapeutic target to inhibit excessive collagen deposition in ECM.

This study reports that the deletion of collagen prolyl 4-hydroxylases *P4ha1* and *P4ha2* alters the site-specific 3-hydroxyproline occupancy percentage in mice skin (Figure 5; Table 4). The elevated levels of 3-hydroxyproline occupancy were detected on COL1A1 P874, COL1A1 P1153, and COL1A2 P803 sites in mice skin upon complete deletion of *P4ha2* (Figure 5; Table 4). These 3-hydroxyprolines are reported to have specificity for the P3H1 enzyme in mice bone collagen 1 (Pokidysheva et al., 2013). Elevation of 3-hydroxylation on these sites hints toward the altered activity of prolyl 3-hydroxylases (P3H1, P3H2, P3H3) upon partial deletion of *P4ha1* and complete deletion of *P4ha2*. These findings show that there is a strong association of P4HA1 and P4HA2 with prolyl 3-hydroxylation activity. However, there is complexity in the connection of P4HA1 and P4HA2 with prolyl 3-hydroxylase activity on these three proline sites. The 3-hydroxylation on COL1A1 P874 and COL1A2 P803 was not changed upon partial deletion of *P4ha2* (*P4ha1* ^*+/+*^; *P4ha2* ^*+/−*^) compared to wild-type (Figure 5; Table 4). This is the first study to identify COL1A1 P874 and COL1A2 P803 sites in the mice skin. These sites are popularly known as A3 sites in COL1A1 and COL1A2. The 3-hydroxylation on COL1A1 P874 and COL1A2 P803 was elevated upon the partial deletion of *P4ha1* and *P4ha2* (*P4ha1* ^*+/−*^; *P4ha2* ^*+/−*^), and 3-hydroxylation was also increased in mice with a complete deletion of *P4ha2* (*P4ha1* ^*+/+*^; *P4ha2−/−*, and *P4ha1* ^*+/−*^; *P4ha2−/−*) compared to the wild-type mice. Among all genotypes, mice with a partial deletion of *P4ha1* and complete deletion of *P4ha2* (*P4ha1* ^*+/−*^; *P4ha2−/−*) have the highest levels of 3-hydroxyproline occupancy on COL1A1 P874 and COL1A2 P803 sites. This means that partial deletion of *P4ha2* cannot alter the 3-hydroxylation activity on COL1A1 P874 and COL1A2 P803 sites in mice skin, but partial deletion of *P4ha1* or complete deletion of *P4ha2* can elevate the 3-hydroxylation occupancy on these two sites.

On the other hand, *P4ha1* deletion and *P4ha2* deletion have different effects on the 3-hydroxyproline occupancy percentage on the COL1A1 P1153 site. This P1153 site, popularly known as the A1 site, is associated with osteogenesis imperfecta. This site has been reported to have specificity for P3H1 in mice tendon, bone, and skin tissues (Pokidysheva et al., 2013). This analysis found that the 3-hydroxylation occupancy percentage on the COL1A1 P1153 site was decreased (∼4% compared to wild-type) upon partial deletion of *P4ha1* and *P4ha2* (*P4ha1* ^*+/−*^; *P4ha2* ^*+/−*^) (Figure 5; Table 4). However, 3-hydroxylation on this site was elevated (∼2% compared to wild-type) in *P4ha1* ^*+/+*^; *P4ha2−/−* mice, and a similar increase was detected in *P4ha1* ^*+/−*^; *P4ha2−/−* mice (Table 4). Partial deletion of *P4ha1* has a different effect on P1153 than it has on COL1A1 P874 and COL1A2 P803 sites. Partial deletion of *P4ha1* with partial deletion *P4ha2* (*P4ha1* ^*+/−*^; *P4ha2* ^*+/−*^) results in decreased 3-hydroxyproline occupancy percentage on the P1153 site (Figure 5). This indicates that partial deletion of *P4ha1* can decrease the 3-hydroxylation level on COL1A1 P1153. However, complete deletion of *P4ha2* can elevate the 3-hydroxylation on P1153 compared to the wild-type. The different effects of *P4ha1* deletion and *P4ha2* deletion hint toward different modes of interaction of P4HA1 and P4HA2 with prolyl 3-hydroxylases.

This study highlights that the effects of *P4ha1* and *P4ha2* deletion are not limited to the proline modifications. This study shows that lysine modifications are also affected by *P4ha1* and *P4ha2* deletion. Despite having enzymatic activities of 4-hydroxylation on proline sites, deletion of *P4ha1* and *P4ha2* can affect the lysine modifications as well. This analysis shows elevated levels of 5-hydroxylysine occupancy on collagen 1 helical cross-linking lysine site (COL1A2 K183) as well as helical non-cross-linking lysine sites (COL1A1 K731 and COL1A2 K315) in mice with a partial deletion of *P4ha1* (*P4ha1* ^*+/−*^; *P4ha2* ± and *P4ha1* ^*+/−*^; *P4ha2−/−*) and mice with a complete deletion of *P4ha2* (*P4ha1* ^*+/+*^; *P4ha2−/−* and *P4ha1* ^*+/−*^; *P4ha2−/−*) compared to the wild-type mice (Figure 6; Table 5). The occupancy percentage of helical non-cross-linking site COL1A2 K315 was increased more than twofold in *P4ha1* ^*+/−*^; *P4ha2−/−* mice compared to the wild-type. Similarly, the levels of occupancy percentages of 5-hydroxylation and O-glycosylation (galactosyl-hydroxylysine) on the COL1A1 K731 site were also increased upon partial deletion of *P4ha1* and complete deletion of *P4ha2*. This study reveals that partial deletion of *P4ha1* and complete deletion of *P4ha2* can elevate the 5-hydroxylysine and O-glycosylation occupancy percentages on helical cross-linking and non-cross-linking lysine sites. These findings indicate that *P4ha1* and *P4ha2* deletion can modulate lysyl 5-hydroxylation activity in collagen 1. Modulation in 5-hydroxylysine occupancy on helical cross-linking sites upon *P4ha1* and *P4ha2* deletion can also have effects on collagen cross-linking.

It has been reported that *P4ha1* ^*+/−*^; *P4ha2−/−* mice have chondrodysplasia and defects in ECM in multiple tissues, while P4ha1^+/+^; P4ha2−/− have minor defects in ECM (Aro et al., 2015; Tolonen et al., 2022; Salo et al., 2024). This study also highlights modulation in modification level on collagen cross-linking sites, which can affect the collagen assembly in the ECM. Collagen cross-linking analysis on *P4ha1*/*P4ha2* deleted or overexpressed mice can delineate the exact effects of *P4ha2* on collagen cross-linking.

**FIGURE 7:**
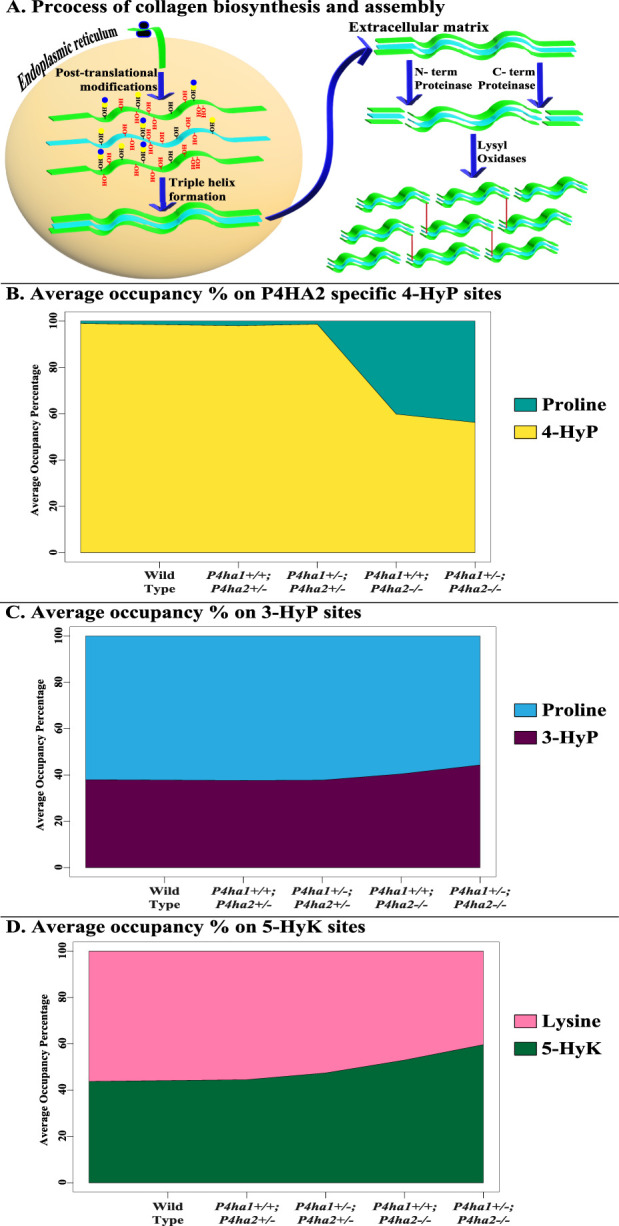
Occurrence of collagen PTMs during biosynthesis and deposition in the ECM. **(A)** shows the biosynthesis and deposition of collagen in the ECM. Newly translated collagen chains enter the endoplasmic reticulum and are heavily modified post-translationally in a site-specific manner by collagen-modifying enzymes. Modified collagen chains form a triple helix, which is transported to the ECM. In the ECM, N- and C-terminal proteinases cleave the pro-peptides of the collagen triple helix. Then, cross-linking and formation of collagen fiber assembly are induced by the activity of lysyl-oxidases. **(B–D)** show the effects of C-P4H deletion on different collagen PTMs. The average of 4-hydroxyproline (4-HyP) occupancy percentages of seven P4HA2-specific sites (Table 3) in wild-type and C-P4H mutant mice is shown in Figure 7B. It was found that average 4-HyP occupancy percentage decreased in mice with a complete deletion of *P4ha2* compared to partial *P4ha1-* or *P4ha2*-deleted and wild-type mice. Interestingly, the average occupancy percentage of three 3-hydroxyproline sites (Table 4) and three 5-hydroxylysine sites (Table 5) was increased (Figures 7C, D) in complete *P4ha2*-deleted mice compared to wild-type and partial *P4ha1-* or *P4ha2*-deleted mice.

This study shows a comprehensive analysis of the effects of partial deletion of *P4ha1* and partial or complete deletion of *P4ha2* on different types of PTMs of mouse skin fibrillar collagen chains. A total of 421 site-specific collagen PTMs are identified in wild-type mice skin fibrillar collagen I (COL1A1 and COL1A2) and collagen III (COL3A1) (Table 1). This study also hints toward the crosstalk between prolyl 4-hydroxylases, prolyl 3-hydroxylation, lysyl 5-hydroxylation, and O-glycosylation in COL1A1 and COL1A2 of mice skin. Deletion of *P4ha1* and *P4ha2* affects the whole collagen PTM networks in mice skin. Some 4-hydroxyproline sites are underhydroxylated in the absence of *P4ha1* and *P4ha2*, while some 3-hydroxyproline, 5-hydroxylysine, and O-glycosylation sites are overmodified in mice skin collagen I (Figure 7). Prolyl 3-hydroxylases, lysyl hydroxylases, and the alpha subunit of prolyl 4-hydroxylases have similarities in the C-terminal dioxygenase domain (Vranka et al., 2004; Pokidysheva et al., 2013). These enzymes also have similar requirements (2-oxoglutarate) for their catalytic activity.

Interestingly, a study on *P3h1* gene knockout has shown that complete deletion of *P3h1* leads to altered lysine modification levels in mice (Pokidysheva et al., 2013). Prolyl 3-hydroxylation is catalyzed by a complex of P3H1, CRTAP, and cyclophilin B. Disruption of this complex by completely deleting cyclophilin B also results in site-specific modulation of lysine modifications (Terajima et al., 2016), indicating that the activities of different collagen-modifying enzymes are somehow interlinked. Three possible reasons for the over-modification of 3-hydroxyproline, 5-hydroxylysine, and O-glycosylation sites in absence of C-P4Hs are: (i) there is direct/indirect crosstalk between different collagen-modifying enzymes, (ii) complete deletion of P4HA2 induces compensative effect on other collagen-modifying enzymes, and (iii) C-P4Hs, along with their catalytic activity, also act as chaperones to facilitate collagen folding and other collagen modify enzymes show compensatory effects in absence of C-P4Hs to modify and fold collagens. This study opens a plethora of opportunities to explore the significance of cooperation between different collagen-modifying enzymes in biosynthesis and the functioning of collagen chains.

## 5 Limitations

This study shows the effects of *P4ha1* and *P4ha2* deletion on site-specific PTMs. However, the effects of deletion of P4HA3 were not studied. The mRNA level expression of C-P4Hs is referred to, but the protein level expressions of C-P4Hs (P4HA1, P4HA2, and P4HA3) in the wild-type and mutant mice skin were not available in this study.

## 6 Future perspectives

This study highlights the crosstalk between different collagen-modifying enzymes and different site-specific collagen PTMs. This crosstalk can be further explored to better understand collagen biosynthesis in wild-type tissues and to therapeutically regulate collagen biosynthesis during fibrosis and cancer progression.

## Data Availability

The original contributions presented in the study are included in the article/Supplementary Material; further inquiries can be directed to the corresponding author.
