# Supplementary material for: Comprehensive analysis of the effects of P4ha1 and P4ha2 deletion on post-translational modifications of fibrillar collagens in mouse skin
Source: Front Cell Dev Biol. 2025 Feb 28;13:1527839. doi: 10.3389/fcell.2025.1527839 (PMC11906473; doi:10.3389/fcell.2025.1527839)
Supplement: Supplementary file 1 [file DataSheet1.pdf]

Supporting Information for

"Comprehensive analysis of effects of *P4ha1* and *P4ha2* deletion on post-translational modifications of fibrillar collagens in mice skin"

Vivek Sarohi [1]

[1] School of Biosciences and Bioengineering (SBB), Indian Institute of Technology (IIT)- Mandi, India

Correspondence – viveksarohi@gmail.com

Table of Contents

S.N.	Title	Page No.
2	Supplementary Figure 1(A) (4-HyP sites)	2-5
3	Supplementary Figure 1(B) (3-HyP sites)	6-8
4	Supplementary Figure 1(C) (HyK sites)	9-10

Supplementary Figure 1: Author identified site-specific collagen post-translational modifications in fibrillar collagen chains (COL1A1, COL1A2, and COL3A1) extracted from wild-type mice skin. Manual analysis of peptide spectrum match (PSM) was performed. PSMs with mass error lower than ±20 ppm and less than 1% FDR were considered for manual analysis. PSMs were annotated using pLabel. Supplementary figure 1(A) (page 2-5) shows PSMs for 4-hydroxyproline sites. Supplementary figure 1(B) (page 6-8) shows PSMs for 3-hydroxyproline sites and Supplementary figure 1(C) (page 9-10) shows PSMs for hydroxylysine sites.

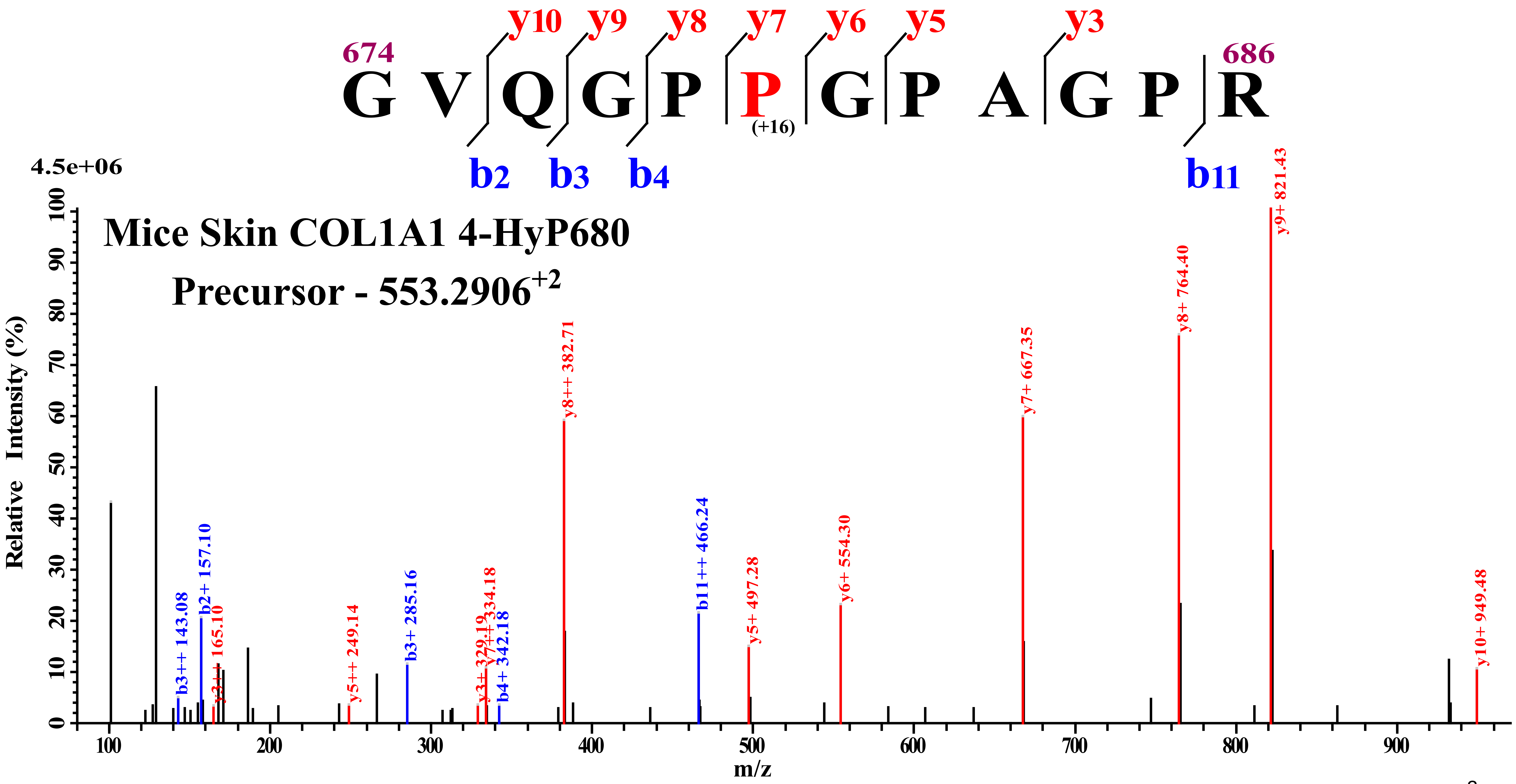
674
G V Q G P P G P A G P R
686
(+16)
y10 y9 y8 y7 y6 y5 y3
b2 b3 b4 b11
4.5e+06
Mice Skin COL1A1 4-HyP680
Precursor - 553.2906+2
Relative Intensity (%)
m/z
b3++ 143.08
b2+ 157.10
y3++ 165.10
y5++ 249.14
b3+ 285.16
y3+ 329.19
y7++ 334.18
b4+ 342.18
y8++ 382.71
b11++ 466.24
y5+ 497.28
y6+ 554.30
y7+ 667.35
y8+ 764.40
y9+ 821.43
y10+ 949.48

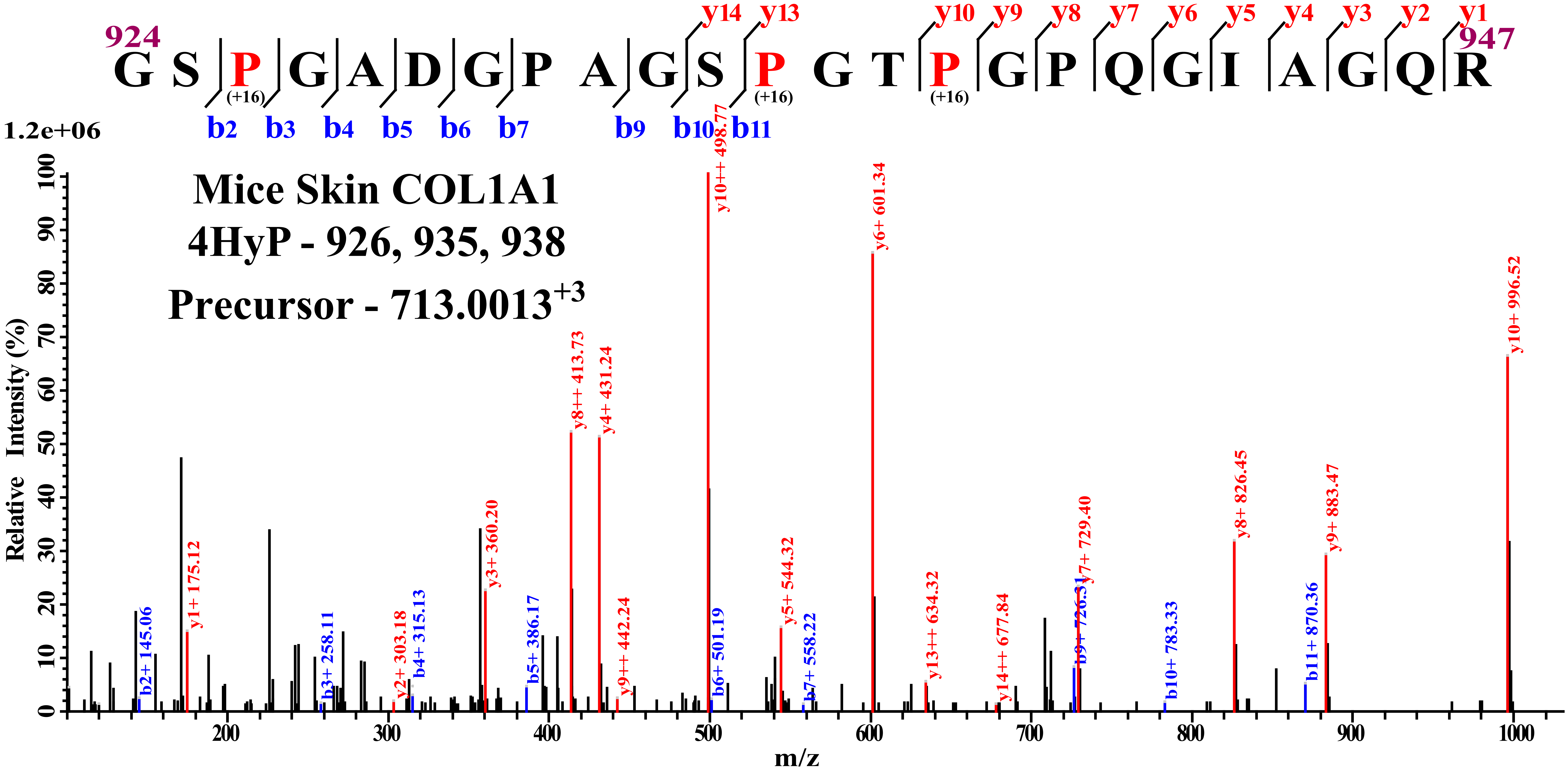

924 G S P(+16) G A D G P A G S P(+16) G T P(+16) G P Q G I A G Q R 947
b2 b3 b4 b5 b6 b7 b9 b10 b11
y14 y13 y10 y9 y8 y7 y6 y5 y4 y3 y2 y1
1.2e+06
Mice Skin COL1A1
4HyP - 926, 935, 938
Precursor - 713.0013+3
Relative Intensity (%)
m/z
b2+ 145.06
y1+ 175.12
b3+ 258.11
y2+ 303.18
b4+ 315.13
y3+ 360.20
b5+ 386.17
y8++ 413.73
y4+ 431.24
y9++ 442.24
y10++ 498.77
b6+ 501.19
y5+ 544.32
b7+ 558.22
y6+ 601.34
y13++ 634.32
y14++ 677.84
y7+ 729.40
b10+ 783.33
y8+ 826.45
b11+ 870.36
y9+ 883.47
y10+ 996.52

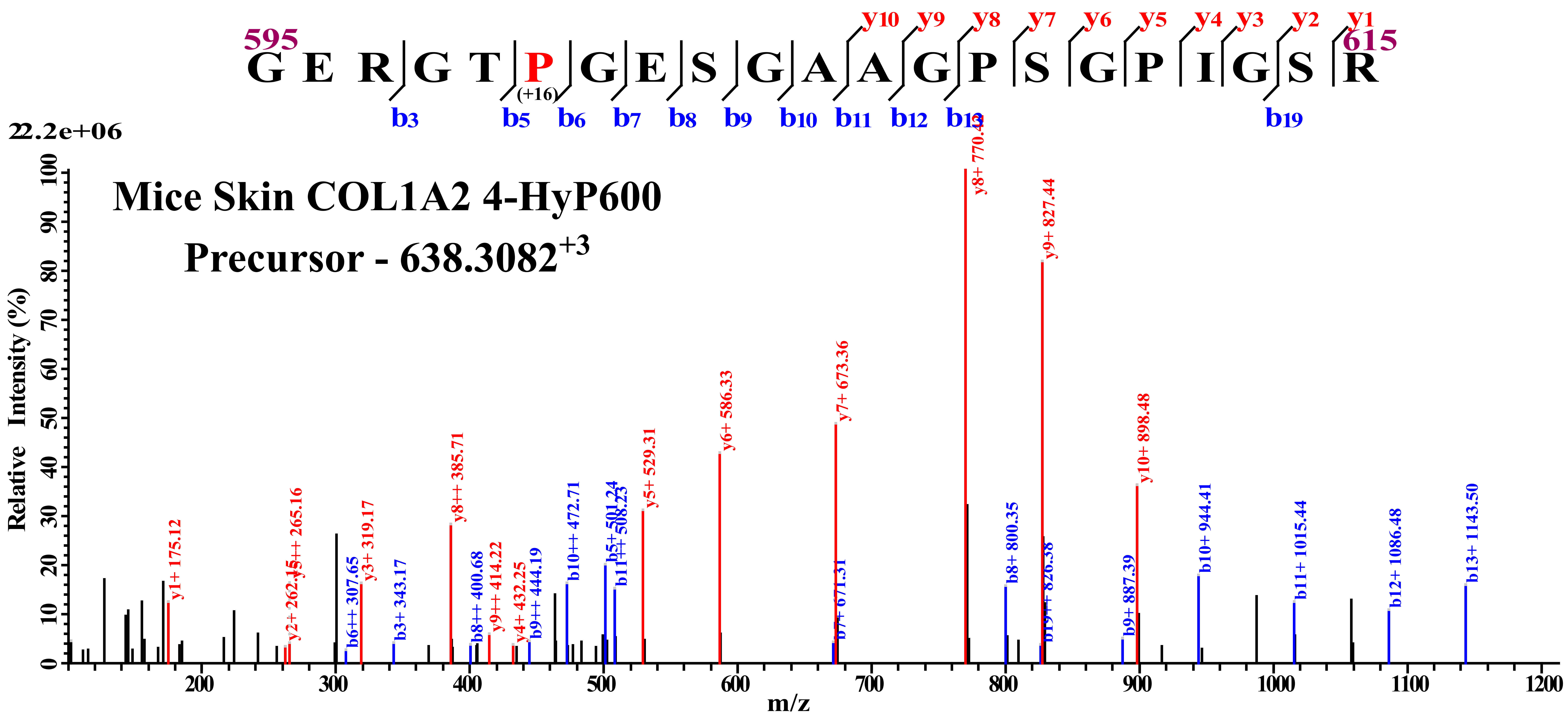

595
615
G E R G T P G E S G A A G P S G P I G S R
(+16)
y10 y9 y8 y7 y6 y5 y4 y3 y2 y1
b3 b5 b6 b7 b8 b9 b10 b11 b12 b13 b19
2.2e+06
Mice Skin COL1A2 4-HyP600
Precursor - 638.3082+3
Relative Intensity (%)
m/z
y8+ 770.4
y9+ 827.44
y7+ 673.36
y6+ 586.33
y10+ 898.48
y5+ 529.31
y8++ 385.71
y1+ 175.12
y3++ 265.16
y2+ 262.15
y3+ 319.17
b6++ 307.65
b3+ 343.17
b8++ 400.68
y9++ 414.22
y4+ 432.25
b9++ 444.19
b10++ 472.71
b5+ 501.24
b11++ 508.23
b7+ 671.31
b8+ 800.35
b19++ 826.38
b9+ 887.39
b10+ 944.41
b11+ 1015.44
b12+ 1086.48
b13+ 1143.50

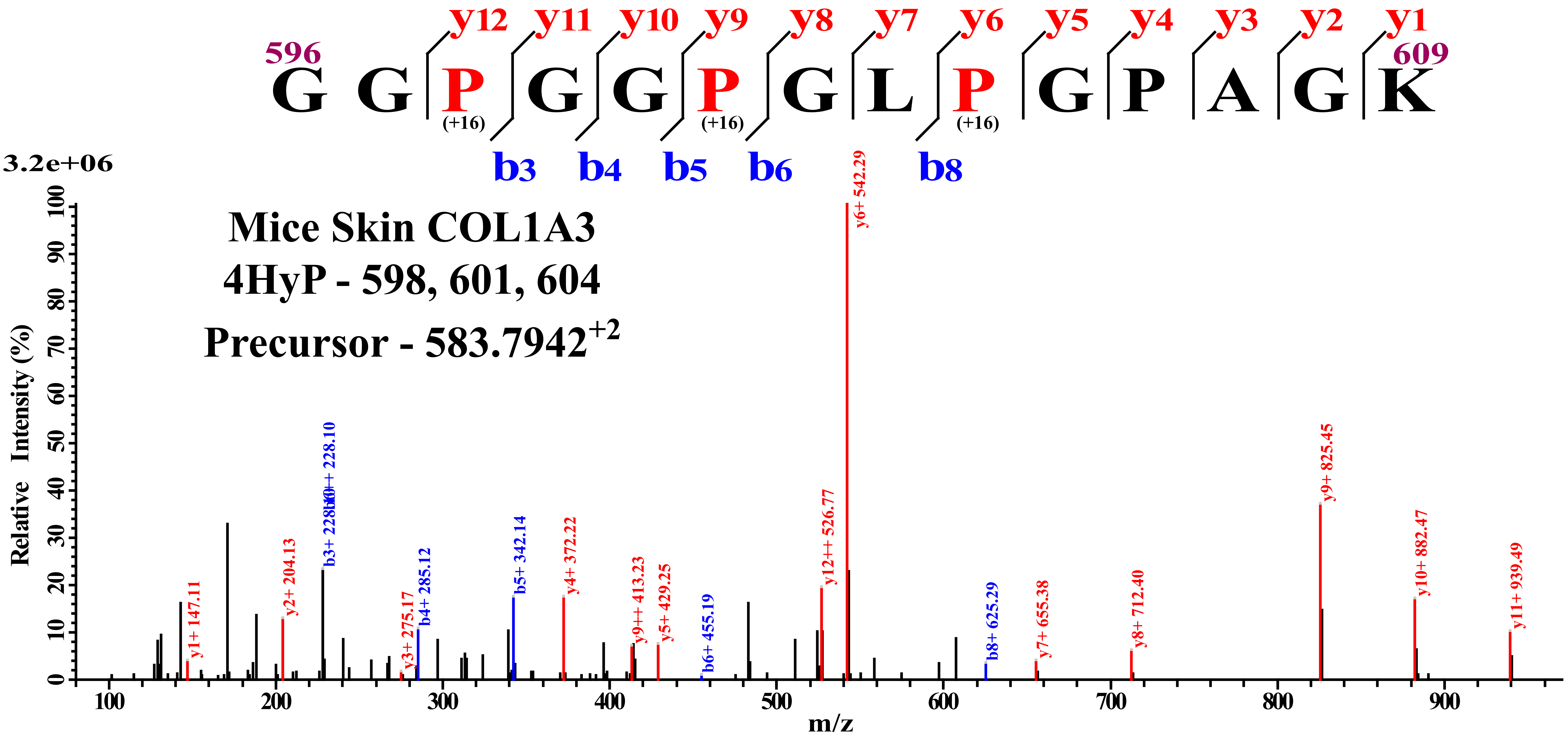

y12 y11 y10 y9 y8 y7 y6 y5 y4 y3 y2 y1
596
G G P G G P G L P G P A G K
609
(+16) (+16) (+16)
b3 b4 b5 b6 b8
3.2e+06
Mice Skin COL1A3
4HyP - 598, 601, 604
Precursor - 583.7942+2
Relative Intensity (%)
m/z
y6+ 542.29
y9+ 825.45
b3+ 228.10
y12++ 526.77
y10+ 882.47
y11+ 939.49
y1+ 147.11
y2+ 204.13
y3+ 275.17
b4+ 285.12
b5+ 342.14
y4+ 372.22
y9++ 413.23
y5+ 429.25
b6+ 455.19
b8+ 625.29
y7+ 655.38
y8+ 712.40

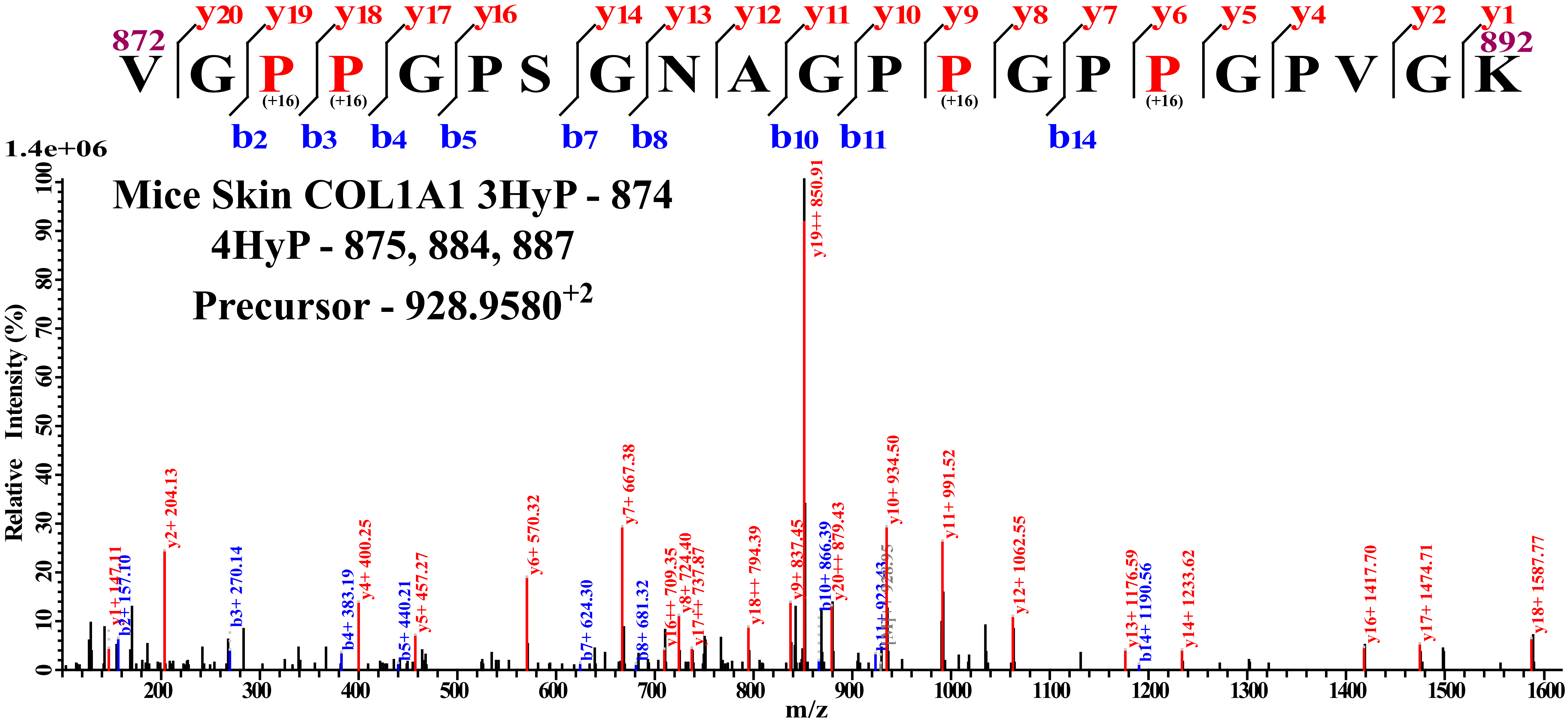

872
V G P P G P S G N A G P P G P P G P V G K
892
(+16) (+16) (+16) (+16)
y20 y19 y18 y17 y16 y14 y13 y12 y11 y10 y9 y8 y7 y6 y5 y4 y2 y1
b2 b3 b4 b5 b7 b8 b10 b11 b14
1.4e+06
Mice Skin COL1A1 3HyP - 874
4HyP - 875, 884, 887
Precursor - 928.9580+2
Relative Intensity (%)
m/z
y1+ 147.11
b2+ 157.10
y2+ 204.13
b3+ 270.14
b4+ 383.19
y4+ 400.25
b5+ 440.21
y5+ 457.27
y6+ 570.32
b7+ 624.30
y7+ 667.38
b8+ 681.32
y16++ 709.35
y8+ 724.40
y17++ 737.87
y18++ 794.39
y9+ 837.45
y19++ 850.91
b10+ 866.39
y20++ 879.43
b11+ 923.43
y10+ 934.50
y11+ 991.52
y12+ 1062.55
y13+ 1176.59
b14+ 1190.56
y14+ 1233.62
y16+ 1417.70
y17+ 1474.71
y18+ 1587.77

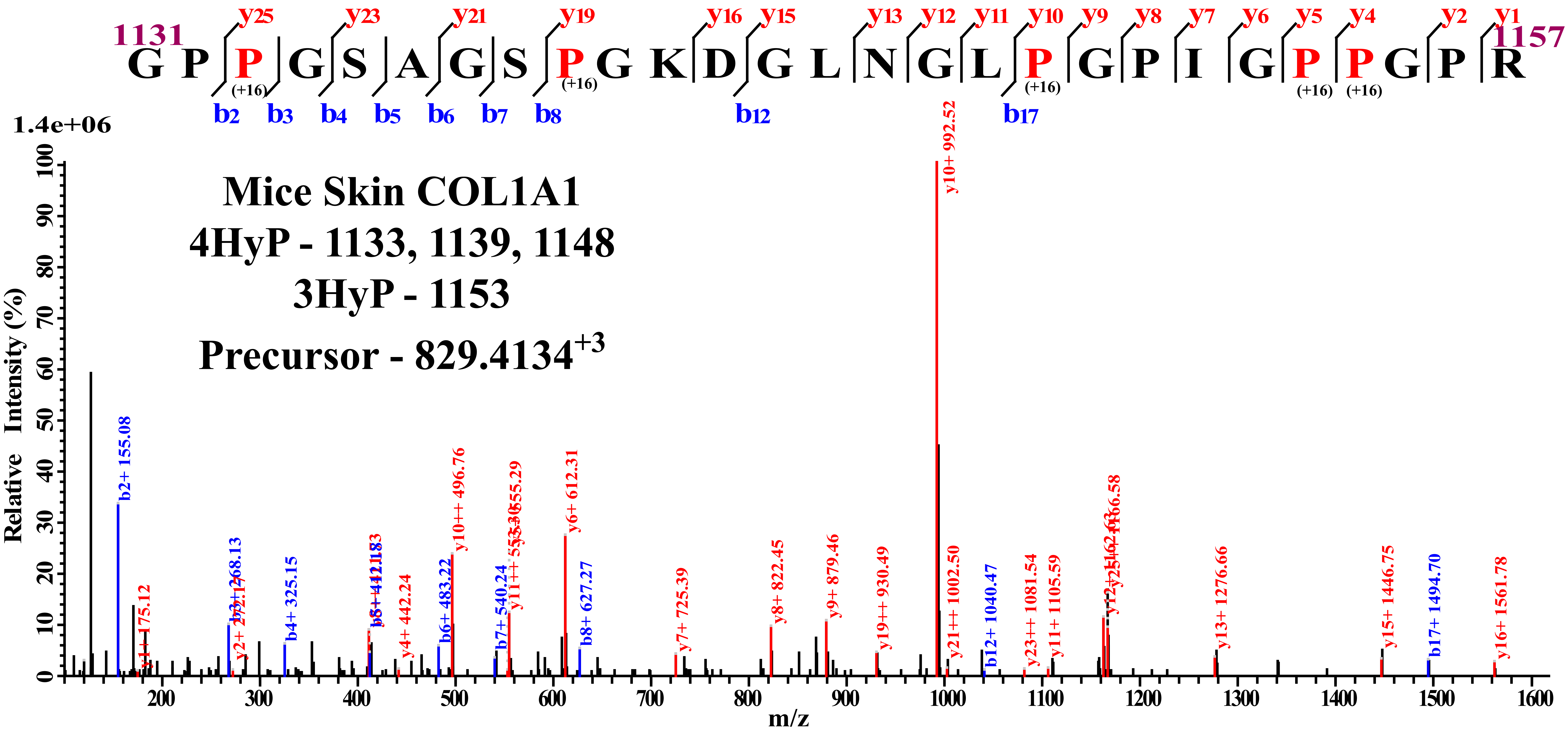

Mice Skin COL1A1
4HyP - 1133, 1139, 1148
3HyP - 1153
Precursor - 829.4134+3
1131
G P P G S A G S P G K D G L N G L P G P I G P P G P R
1157
(+16)
y25 y23 y21 y19 y16 y15 y13 y12 y11 y10 y9 y8 y7 y6 y5 y4 y2 y1
b2 b3 b4 b5 b6 b7 b8 b12 b17
1.4e+06
Relative Intensity (%)
m/z
b2+ 155.08
y1+ 175.12
b3+ 268.13
y2+ 272.17
b4+ 325.15
y4+ 442.24
b6+ 483.22
y10++ 496.76
b7+ 540.24
y6+ 612.31
b8+ 627.27
y7+ 725.39
y8+ 822.45
y9+ 879.46
y19++ 930.49
y10+ 992.52
y21++ 1002.50
b12+ 1040.47
y23++ 1081.54
y11+ 1105.59
y13+ 1276.66
y15+ 1446.75
b17+ 1494.70
y16+ 1561.78

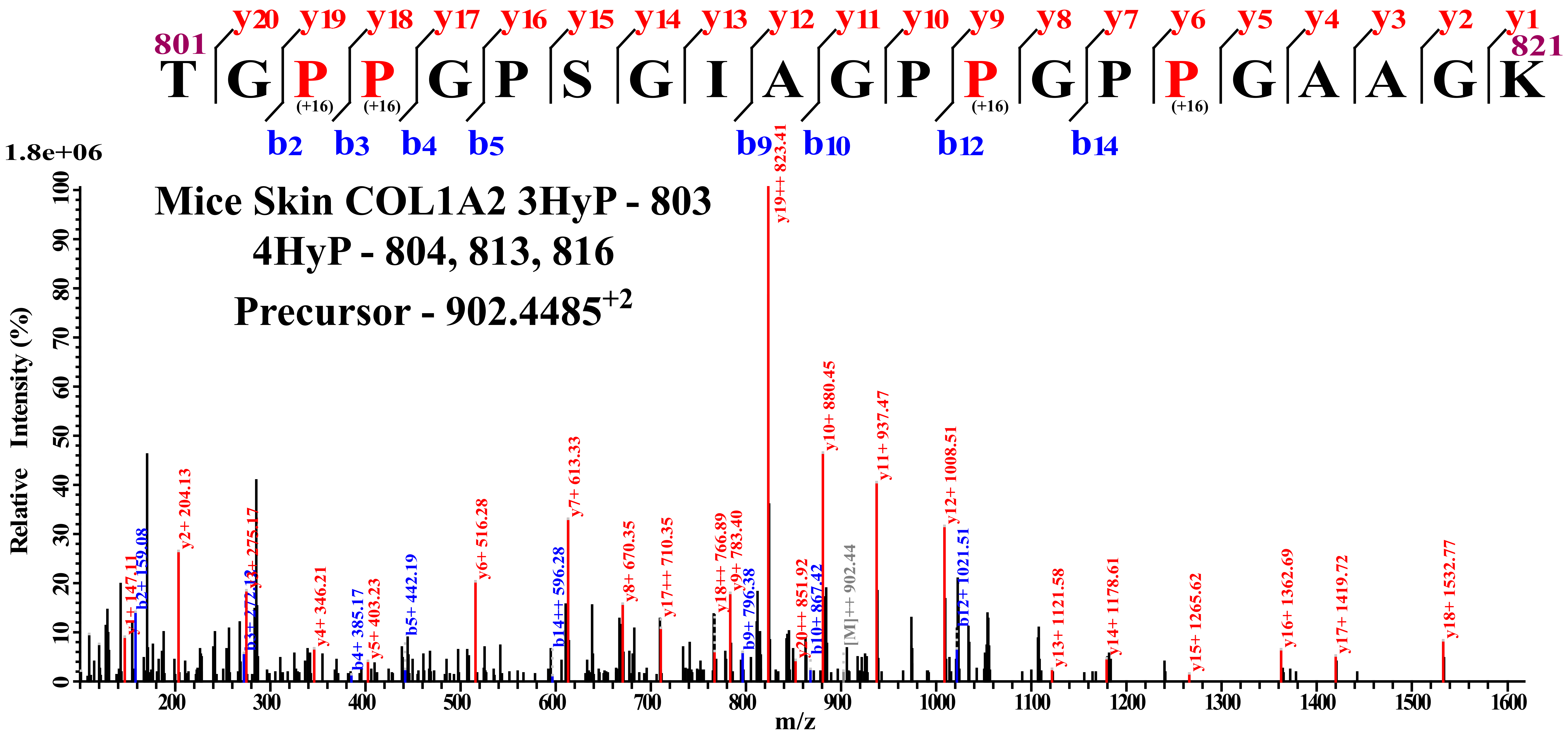
801
T G P P G P S G I A G P P G P P G A A G K
821
(+16)
(+16)
(+16)
(+16)
y20 y19 y18 y17 y16 y15 y14 y13 y12 y11 y10 y9 y8 y7 y6 y5 y4 y3 y2 y1
b2 b3 b4 b5 b9 b10 b12 b14
1.8e+06
Mice Skin COL1A2 3HyP - 803
4HyP - 804, 813, 816
Precursor - 902.4485+2
Relative Intensity (%)
m/z
y19++ 823.41
y10+ 880.45
y11+ 937.47
y12+ 1008.51
y7+ 613.33
y6+ 516.28
y2+ 204.13
b2+ 159.08
y1+ 147.11
b3+ 277.13
y3+ 275.17
y4+ 346.21
b4+ 385.17
y5+ 403.23
b5+ 442.19
b14++ 596.28
y8+ 670.35
y17++ 710.35
y18++ 766.89
y9+ 783.40
b9+ 796.38
y20++ 851.92
b10+ 867.42
[M]++ 902.44
b12+ 1021.51
y13+ 1121.58
y14+ 1178.61
y15+ 1265.62
y16+ 1362.69
y17+ 1419.72
y18+ 1532.77

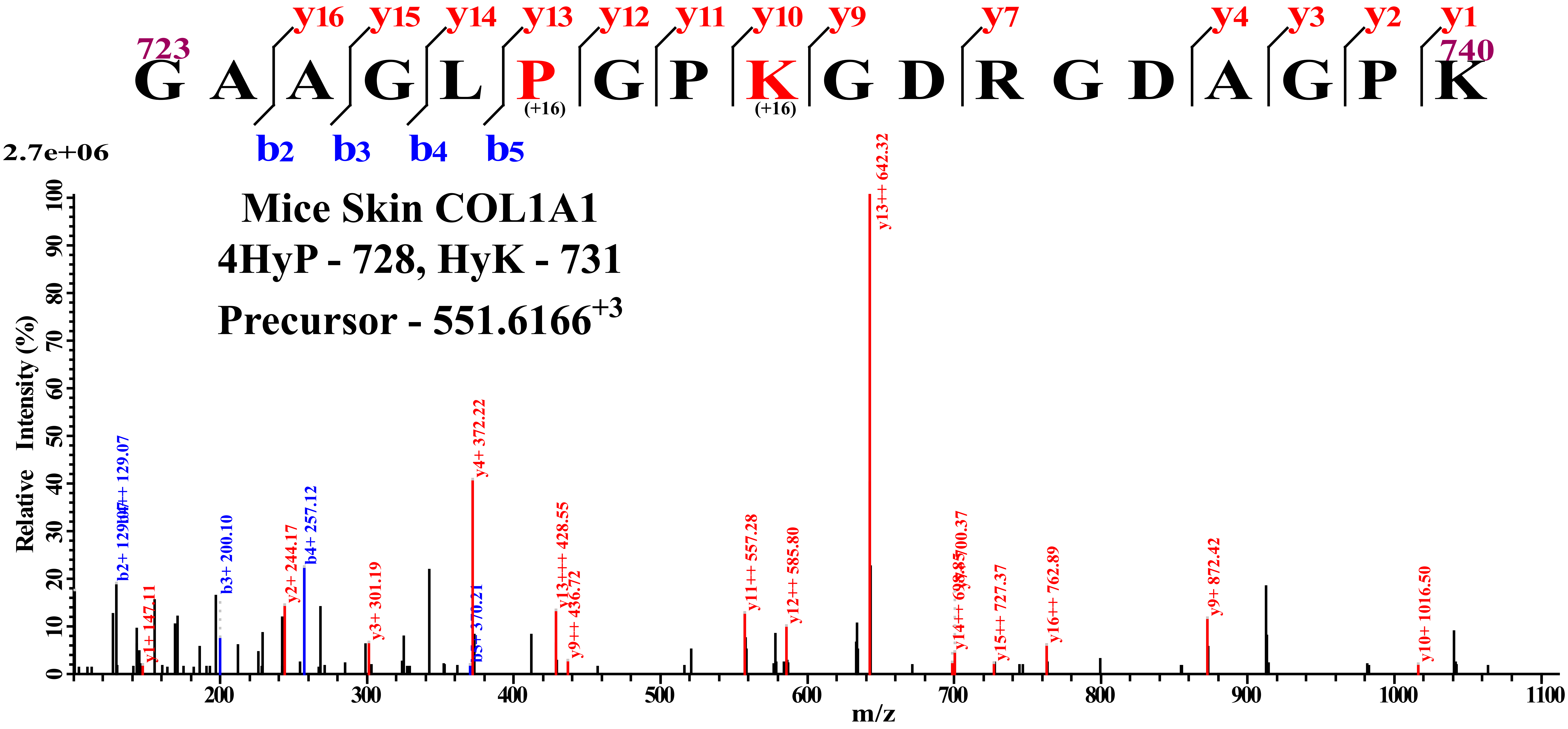
Mice Skin COL1A1
4HyP - 728, HyK - 731
Precursor - 551.6166+3
723
G A A G L P G P K G D R G D A G P K
740
(+16)
(+16)
y16 y15 y14 y13 y12 y11 y10 y9 y7 y4 y3 y2 y1
b2 b3 b4 b5
2.7e+06
Relative Intensity (%)
m/z
b2+ 129.07
b3+ 200.10
b4+ 257.12
b5+ 370.21
y1+ 147.11
y2+ 244.17
y3+ 301.19
y4+ 372.22
y13+++ 428.55
y9++ 436.72
y11++ 557.28
y12++ 585.80
y13++ 642.32
y14++ 700.37
y15++ 727.37
y16++ 762.89
y9+ 872.42
y10+ 1016.50

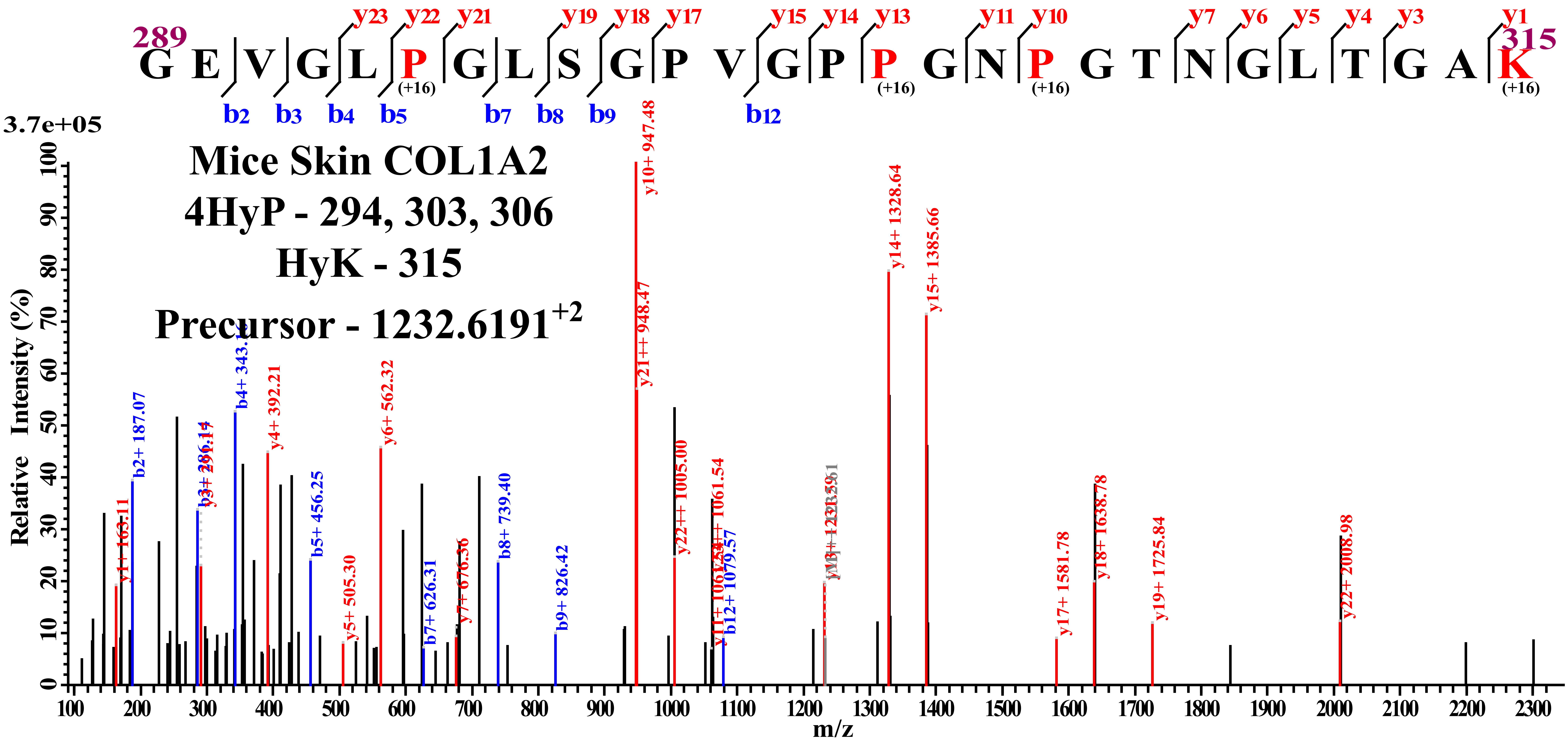

Mice Skin COL1A2
4HyP - 294, 303, 306
HyK - 315
Precursor - 1232.6191+2
289
G E V G L P G L S G P V G P P G N P G T N G L T G A K
315
(+16)
y23 y22 y21 y19 y18 y17 y15 y14 y13 y11 y10 y7 y6 y5 y4 y3 y1
b2 b3 b4 b5 b7 b8 b9 b12
3.7e+05
Relative Intensity (%)
m/z
y10+ 947.48
y21++ 948.47
y14+ 1328.64
y15+ 1385.66
y6+ 562.32
y4+ 392.21
b4+ 343.1
b2+ 187.07
b3+ 286.14
y3+ 291.17
y1+ 163.11
b5+ 456.25
y5+ 505.30
b7+ 626.31
y7+ 676.36
b8+ 739.40
b9+ 826.42
y22++ 1005.00
y11+ 1061.54
b12+ 1079.57
y17+ 1581.78
y18+ 1638.78
y19+ 1725.84
y22+ 2008.98
